# A Possible Unmet Need: Pneumococcal Vaccination in the Workplaces—A Systematic Review of Invasive Pneumococcal Disease Among Shipyard Workers

**DOI:** 10.3390/vaccines14050437

**Published:** 2026-05-13

**Authors:** Matteo Riccò, Luca Pipitò, Claudio Costantino, Silvio Tafuri, Chiara Noviello, Marco Bottazzoli, Paolo Manzoni, Daniel Fiacchini, Marco Falcone, Pasquale Gianluca Giuri, Davide Gori, Antonio Cascio

**Affiliations:** 1 AUSL–IRCCS di Reggio Emilia, Servizio di Prevenzione e Sicurezza Negli Ambienti di Lavoro (SPSAL), Local Health Unit of Reggio Emilia, 42122 Reggio Emilia, Italy; 2 Infectious and Tropical Diseases Unit, Department of Health Promotion, Mother and Child Care, Internal Medicine and Medical Specialties, “G D’Alessandro”, University of Palermo, AOUP P. Giaccone, 90127 Palermo, Italy; 3 Department of Health Promotion Sciences, Maternal and Infant Care, Internal Medicine and Medical Specialties (PROMISE) “G. D’Alessandro”, University of Palermo, 90127 Palermo, Italy; claudio.costantino01@unipa.it; 4 Department of Interdisciplinary Medicine, Aldo Moro University of Bari, 70121 Bari, Italy,; 5 Department of Otorhinolaryngology, ASUIT Trento, 38122 Trento, Italy; 6 SCDU Pediatria e Neonatologia, Ospedale Degli Infermi, Ponderano (BI), Dipartimento Materno-Infantile ASLBI, Università di Torino, 10124 Torino, Italy; 7 AST Ancona, Prevention Department, UOC Sorveglianza e Prevenzione Malattie Infettive e Cronico Degenerative, 61100 Ancona, Italy; 8 Department of Clinical and Experimental Medicine, University of Pisa, 56126 Pisa, Italy; 9 Department of Medicine and Diagnostics, AUSL di Parma, 43100 Parma, Italy; 10 Department of Biomedical and Neuromotor Sciences, University of Bologna, 40126 Bologna, Italy; davide.gori4@unibo.it

**Keywords:** *Streptococcus pneumoniae*, invasive pneumococcal disease, occupational disease, pneumococcus, prevalence

## Abstract

**Background**: Workplace-related outbreaks of invasive pneumococcal disease (IPD) have been increasingly reported among shipyard workers, yet their epidemiological and clinical features remain incompletely characterized. This systematic review and meta-analysis aimed to synthesize available evidence on IPD outbreaks in shipyard settings. **Methods**: A systematic search of PubMed/MEDLINE, Scopus, EMBASE, and medRxiv was conducted up to March 2026. Observational studies reporting IPD outbreaks in shipyards were included. Pooled incidence rates and clinical outcomes were estimated using random-effects models, with heterogeneity assessed by I^2^ statistics. Risk of bias was evaluated using the Newcastle–Ottawa Scale. **Results**: Eight studies describing six outbreaks across four European countries (France, Norway, Northern Ireland, Finland; 2015–2025) were included, encompassing 131 cases among 35,623 workers. The pooled incidence was 368.9 cases per 100,000 workers with an attack rate of 2.36 per 1000 person-months for total cases, compared to 200.49 cases per 100,000 workers (95%CI 103.54–387.85) and 1.10 cases per 1000 person-months (95% CI 0.17–2.03) for laboratory confirmed cases, with considerable heterogeneity across studies. Most cases occurred in men (97.7%), with the median age ranging from 39 to 48 years. Hospitalizations occurred in 79.1% of cases, intensive care unit admission in 13.7%, and the case fatality ratio was 0.8%. Serotype 4 accounted for 67.2% of characterized isolates. Occupational exposures and shared accommodation may have contributed to transmission, although this could not be formally assessed. **Conclusions**: IPD outbreaks in shipyard settings are characterized by high incidence but relatively favorable outcomes, likely reflecting workforce demographics. However, considerable heterogeneity and methodological limitations across studies constrain the interpretation of pooled estimates. Preventive strategies, including vaccination and workplace-targeted interventions, should be considered as plausible public health measures, with a proactive role for occupational health services.

## 1. Introduction

*Streptococcus pneumoniae* (pneumococcus) is a highly invasive, Gram-positive, extracellular bacterial pathogen [1,2], which, based on the capsular antigens, the main virulence factor, is classified into more than 90 distinct serotypes [3,4,5].

Pneumococcus causes a wide range of human diseases, from non-invasive infections of the respiratory mucosa to invasive conditions such as otitis media, sinusitis, conjunctivitis and community-acquired pneumonia [6,7,8,9], which may in turn progress to invasive infections. In these cases, the pathogen breaches the body’s defenses and can be identified from normally sterile body sites. Collectively, these infections are defined as invasive pneumococcal diseases (IPD), and include bacteremia, sepsis, meningitis, osteomyelitis, as well as less frequent infections such as periorbital cellulitis, osteomyelitis, endocarditis, pericarditis, peritonitis, pyogenic arthritis, soft tissue infections and neonatal septicemia [10].

Invasive pneumococcal diseases are considered leading contributors to the global burden of vaccine-preventable diseases [4,5,11], in terms of both morbidity and mortality. It is estimated to account for approximately 8.1 million disability-adjusted life years (DALYs) lost annually [6,9,11], and around 830,000 deaths, most of which are associated with pneumococcal pneumonia, corresponding to approximately 40.3 million life-years lost [6,7,8,12]. More precisely, pneumococcal pneumonia has historically been recognized as the single leading cause of mortality from lower respiratory infections, accounting for more deaths than all other pathogens combined (e.g., 76,000 deaths for respiratory syncytial virus; 58,000 deaths for seasonal influenza; and 48,000 deaths for *Haemophilus influenzae* type b) [13]. In the United States alone, over the past decade, pneumococcal pneumonia has accounted for 12% to 13% of all hospitalized pneumonia cases, corresponding to approximately 225,000 hospitalizations, including around 30,000 cases of IPD [2,14].

Pneumococcal infections and IPD can affect people of all ages, but in most high-income countries, they typically exhibit a U-shaped curve [2,10,12,15,16], with the highest incidence reported from older adults and infants [9,12,13,16]. For example, according to 2022 report from European Center for Disease Prevention and Control (ECDC), notification rates for IPD in European countries ranged from 12.6 cases per 100,000 population among adults aged ≥ 65 years to 13.4 per 100,000 population among infants under one year of age, with the lowest rates observed in the 15–24 year age group (i.e., 0.8 cases per 100,000 population) [10]. Nonetheless, the same data indicate that IPD cases in working-age groups (25–64 years) represent a substantial proportion of total incident cases, accounting for approximately 37% of all reported cases, with a case fatality ratio (CFR) ranging from 19% to 24% [10,17,18].

An increased risk of developing IPD, as well as more severe outcomes, has been associated with several clinical conditions, including smoking, chronic heart, lung, liver, or renal disease (particularly chronic kidney disease), as well as conditions leading to impaired immune function (e.g., transplant recipients, immunodeficiency, hematological malignancies, and functional or anatomic asplenia), and diabetes [4,5,9,12,16,19]. Moreover, several studies have suggested that certain occupational exposures may increase the occurrence of pneumococcal pneumonia and IPD [20,21,22,23,24,25,26,27,28,29,30,31,32,33,34]. In particular, a previous systematic review on work-related pneumococcal diseases estimated an occupational burden of 10.0%, with an attributable fraction ranging from 38% and 70% [35], and a substantially increased risk among welders and individuals exposed to metal and welding fumes [24,25,28,36,37,38]. In this regard, a systematic review from Riccò et al. 2023 [38] reported that welders have a significantly increased likelihood of developing IPD compared with non-welders (odds ratio [OR] 2.59, 95% CI 2.00–3.35, I^2^ = 0%, *p* = 0.58), as well as a higher risk of mortality from IPD (standardized mortality ratio [SMR] 2.42, 95% CI 1.96–2.99, I^2^ = 0%, *p* = 0.58).

Among the occupational exposures and groups assessed [38], shipyard environments have been identified as among the highest-risk settings for IPD [39,40,41], with an increasing number of outbreaks reported across multiple countries. The reasons underlying the substantial burden of IPD in shipyard settings currently remain largely unclear, but the observed risk is likely driven by a combination of occupational exposures (e.g., metal and welding fumes) and structural factors, including crowding and shared accommodation. Given the limited cumulative evidence in these specific settings and the large populations potentially affected in these workplace centers, this systematic review was undertaken as an update of our previous systematic review on IPD and welding/metal fumes [38], with a specific focus on outbreaks in shipyard settings. By systematically synthesizing outbreak evidence, this study seeks to inform risk assessment and support the development of targeted preventive strategies in high-risk occupational settings.

## 2. Materials and Methods

### 2.1. Study Selection, Inclusion and Exclusion Criteria

The present review was originally designed as an update of a previous review on the occurrence of IPD among workers exposed to metal and welding fumes [38], and was conducted in accordance with the “Preferred Reporting Items for Systematic Reviews and Meta-Analyses” (PRISMA) guidelines [42] (PROSPERO registration number CRD42023404926). A Patient/Population/Problem; Exposure/Context; Outcome strategy [43,44] was designed, given the descriptive nature of outbreak reports and the absence of a consistent comparator. In fact, the review was designed in order to systematically characterize reported outbreaks of pneumococcal disease in shipyard settings, describing their epidemiological features, clinical burden, and associated occupational and environmental conditions, with particular attention to exposure to metal and welding fumes. Relevant studies were identified through three scholarly databases (PubMed/MEDLINE, Scopus, and EMBASE) and the pre-print repository medrxiv.org up to 10 March 2026, without backward chronological restrictions on publication date.

The search strategy resulted from the combination of the search strings reported in Table 1, respectively, for PubMed (through Medical Subject Heading [MeSH] terms), MedRxiv, Scopus and EMBASE:

Retrieved records were handled using the references management software Mendeley Reference Manager 2.144.0 (2026 Elsevier Ltd., London, UK). Titles and abstracts were independently screened by two authors (CN and MB). Only original observational studies reporting documented outbreaks and providing data on the number of diagnosed pneumococcal infections were initially considered. Articles deemed relevant to the study objectives were then assessed in full text to determine whether they met the following inclusion criteria:

- Providing a clear case definition for IPD;
- Providing the exact timeframe of the outbreak;
- Reporting the crude number of assessed cases of IPD: generic diagnoses such as “respiratory infections” or “pneumonia” not otherwise specified were removed from the analyses;
- Reporting the total number of exposed workers from shipyards;
- Reporting the settings of occupational exposure, specifically focusing on the exposure to welding and metal fumes, assessed through job titles (e.g., welders) or job exposure matrix;

In addition to studies not meeting the inclusion criteria, we excluded: (a) articles written in languages other than Italian, German, Swedish, English, French, Spanish and Portuguese (i.e., the languages spoken by the investigators), (b) secondary studies and derived studies (i.e., review articles, meta-analyses); (c) meeting reports and conference abstracts.

### 2.2. Data Extraction

Data extracted included:

1. Settings of the study (country, time, occupational settings);
2. Time interval between the first and the last case (where available).
3. Number of cases (i.e., IPD, admissions to intensive care units [ICU], deaths);
4. Number of workers from the involved shipyard;
5. Number and demographics of pneumococcal infection cases (where available), including age, gender, comorbidities, accommodation conditions (i.e., living alone or with roommates), smoking history;
6. Pneumococcal serogroups and genotypes (where available);
7. Proportion of cases exposed to welding and metal fumes;
8. Vaccination status of included subjects;
9. Outbreak management strategies, i.e., mass vaccination campaigns using pneumococcal conjugate vaccine (PCV), and/or pneumococcal polysaccharide vaccine (PPSV23), and/or by pneumococcal delivery of antibiotic treatment to shipyard workers.

Each outbreak was used as the unit of analysis. When multiple reports described the same outbreak, data were consolidated into a single outbreak-level record by integrating complementary information across sources.

### 2.3. Qualitative Assessment

The methodological quality of the included studies was assessed using the Newcastle–Ottawa Scale (NOS; score range 0–9) [45], a validated tool for evaluating risk of bias in observational studies and widely adopted in systematic reviews [46]. The NOS examines bias across four domains—selection, performance, detection, and information bias—with criteria specifically developed for case–control and cohort designs [47]. In the absence of a dedicated framework for outbreak investigations, the cohort version of the Newcastle–Ottawa Scale (NOS) was applied to all included studies, complemented by an adapted version of the “Outbreak Reports and Intervention studies Of Nosocomial infection (ORION)” statement to address outbreak-specific reporting characteristics [48,49]. Two investigators independently evaluated each eligible study and summarized potential limitations. Discrepancies were resolved through discussion and consensus, with adjudication by a third reviewer when agreement could not be reached.

### 2.4. Data Analysis

#### 2.4.1. Descriptive Analysis

All studies ultimately retained for the present systematic review and meta-analysis were initially synthesized descriptively through the calculation of crude incidence estimates for pneumococcal detection and corresponding attack rates across the shipyard working population. Each outbreak was included only once in the calculation of crude estimates as well as in the meta-analysis to avoid double-counting, with preference given to the most complete and recent report.

The incidence of IPD was calculated both as cumulative incidence per 100,000 workers and as incidence rates per 1000 person-months. For each outbreak, the population at risk was defined as the number of workers reported to be present in the shipyard during the outbreak period, as provided by the original reports. The duration of the outbreak was defined as the time interval (in days) between the first and the last reported case. Person-time at risk was then estimated by multiplying the workforce size by the outbreak duration, expressed in months (days/30), assuming a constant workforce size over the outbreak period. Incidence rates per 1000 person-months were calculated accordingly. When available, both total reported cases and laboratory-confirmed cases were used as numerators, with the latter included in sensitivity analyses. For the aims of the present study, we implicitly assumed that the entire workforce was at risk for the full duration of the outbreak. Given the lack of detailed data on individual exposure time, worker turnover, and task-specific risk, these estimates should be interpreted as proxies of the actual person-time at risk.

#### 2.4.2. Meta-Analysis

Incidence rates of IPD, together with the proportions of hospitalizations, ICU admissions, and case fatality ratios, were pooled using a random-effects meta-analytic framework based on inverse-variance weighting. The meta-analysis was performed as an exploratory analysis to summarize patterns across outbreaks. Between-study variance (τ^2^) was estimated by maximum likelihood methods. Prior to pooling, proportions were transformed using the Freeman–Tukey double arcsine transformation, which is generally preferred over logit transformation when dealing with small and heterogeneous samples because it stabilizes variances and allows inclusion of studies with proportions close to 0 or 1 without requiring continuity corrections. This approach was particularly relevant given the small number of events and the presence of extreme proportions in some outbreaks. In contrast, the logit transformation may be less stable in the presence of sparse data and typically requires continuity corrections when zero events are observed [50]. No zero-event corrections were required, as only outbreak-based datasets were included, inherently precluding zero-event studies for the outcomes considered.

Summary estimates and corresponding 95% confidence intervals were derived using the conservative Clopper–Pearson approach [51]. A random-effects model was selected over a fixed-effects model owing to its greater appropriateness for meta-analyses involving a limited number of studies and substantial between-study heterogeneity [52,53]. Between-study variance was specifically estimated using restricted maximum likelihood (REML), which, unlike the DerSimonian–Laird estimator, does not depend on the pooled effect size and is therefore considered less susceptible to residual bias [54].

#### 2.4.3. Heterogeneity

Between-study heterogeneity (i.e., variability in effect estimates across included studies) was quantified using the I^2^ statistic, which represents the proportion of total variation attributable to true between-study differences rather than sampling error [55,56]. As noted by Hippel et al. [56], point estimates of the I^2^ statistic derived from small meta-analyses may provide unreliable estimates of the true extent of heterogeneity. Accordingly, 95% confidence intervals for I^2^ were also calculated and reported. In line with current recommendations, heterogeneity was categorized as low (≤25%), moderate (26–49%), substantial (50–75%), or considerable (>75%) [46,56,57].

#### 2.4.4. Sensitivity Analysis

Sensitivity analyses were undertaken to assess the robustness of the pooled estimates to uncertainty in the underlying data. Specifically, a leave-one-out approach was applied, whereby pooled incidence and attack rate estimates and corresponding I^2^ statistics were recalculated after sequential exclusion of each study.

#### 2.4.5. Publication Bias

Because meta-analytic findings depend on the completeness and quality of the underlying evidence base, publication bias—arising from the preferential inclusion of published studies—can introduce systematic distortion in pooled estimates and compromise the validity of the conclusions [58]. Potential publication bias was initially explored through visual inspection of funnel plots, in which study-specific effect sizes were plotted against their standard errors. In the absence of bias, a symmetrical distribution around the pooled estimate is expected, whereas asymmetry may suggest the presence of small-study effects or selective reporting. Funnel plot asymmetry was formally assessed using Egger’s regression test in analyses including three or more studies, based on a weighted linear regression of effect estimates on their standard errors [42,59,60].

A quantitative assessment of publication bias was further undertaken using the Luis Furuya-Kanamori (LFK) index in conjunction with Doi plots. Doi plots provide a graphical approach to detecting small-study effects and potential publication bias by plotting standardized effect sizes against a measure of precision; symmetry around the central axis is expected in the absence of bias. Compared with conventional funnel plots, Doi plots have been shown to offer greater sensitivity in identifying asymmetry, particularly when interpreted alongside the LFK index, which quantifies the degree of asymmetry. LFK values below 1 were considered indicative of no or minimal asymmetry, values between 1 and 2 of minor asymmetry, and values greater than 2 of major asymmetry, suggesting potential publication bias. In addition, small-study effects were explored using radial plots, which allow comparison of study estimates with differing levels of precision [61].

A *p*-value < 0.05 was considered statistically significant for both publication bias and small-study effects.

#### 2.4.6. Software

Mendeley Reference Manager (version 2.121.0; Mendeley Ltd.; New York, NY, USA) was employed to manage suitable articles and perform screening and rating procedures. Calculations required by meta-analysis were performed using R (version 4.4.1) [62] and RStudio (version 2024.04.2 Build 764; Posit Software, PBC; Boston, MA, USA) software using the packages *meta* (version 7.0), *fmsb* (version 0.7.5), *epiR* (version 2.0.63), and *robvis* (version 0.3.0). Plots were calculated using the R packages *ggplot2* (version 3.4.3), *ggpubr* (version 0.6.0), and GraphPad Prism, version 10.0 (GraphPad Software LLC, Boston, MA, USA).

## 3. Results

### 3.1. Summary of Retrieved Studies

**Figure 1:**
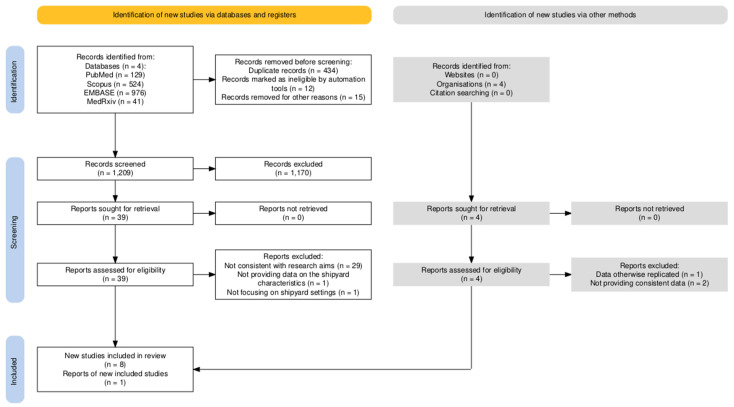
Flowchart of included studies.

The results of the search strategy are summarized in Figure 1. As shown in Table 1, a total of 1670 articles were retrieved (i.e., PubMed 7.7%; Scopus 31.4%; EMBASE 58.4%, medRxiv 2.5%).

Among retrieved entries, 434 (26.0%) were cross-replicated across the searched platforms and were therefore removed from the analyses. A total of 15 articles (0.9%) were excluded because they were published in languages not understood by the study Authors, and an additional 12 records (0.7%) were excluded due to insufficient methodological transparency. Among the remaining screened records (1209, i.e., 72.4% of the original sample), a total of 39 (2.3%) were considered consistent with the research question by title screening and abstract screening.

Among the retrieved articles, 29 were considered not consistent with the research question by full-text screening, including high-quality cross-sectional and case-control studies from Newhouse et al. 1985 [41], Coggon et al. 1994 [26], Palmer et al. 2003 [31], Palmer et al. 2009 [33], Palmer et al. 2013 [32], Wong et al. 2010 [24], Torén et al. 2011 [23], Sen et al. 2012 [27], Palmer et al. 2012 [25], Suri et al. 2016 [34], Torén et al. 2020 [20], Torén et al. 2022 [21,22], and Torén et al. 2023 [63]. Two additional entries from conference proceedings were similarly excluded as not consistent with the research strategy [64,65].

The report from Gladstone et al. 2022 [66] was also excluded, as it focused on serotype characteristics and did not provide clinical or occupational data. Two case series were subsequently removed from the analysis because they did not report data on the exposed population in the source shipyard [67], or were not consistent with the shipyard settings [68].

**Figure 2:**
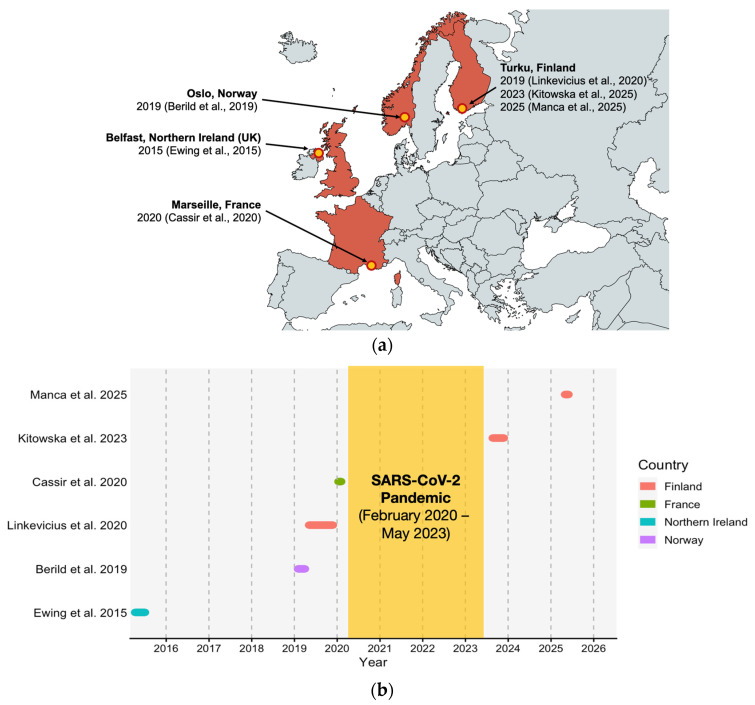
Timeline (**a**) and location (**b**) of shipyard outbreaks included in the present systematic review [29,40,69,70,71,72,73].

**Figure 3:**
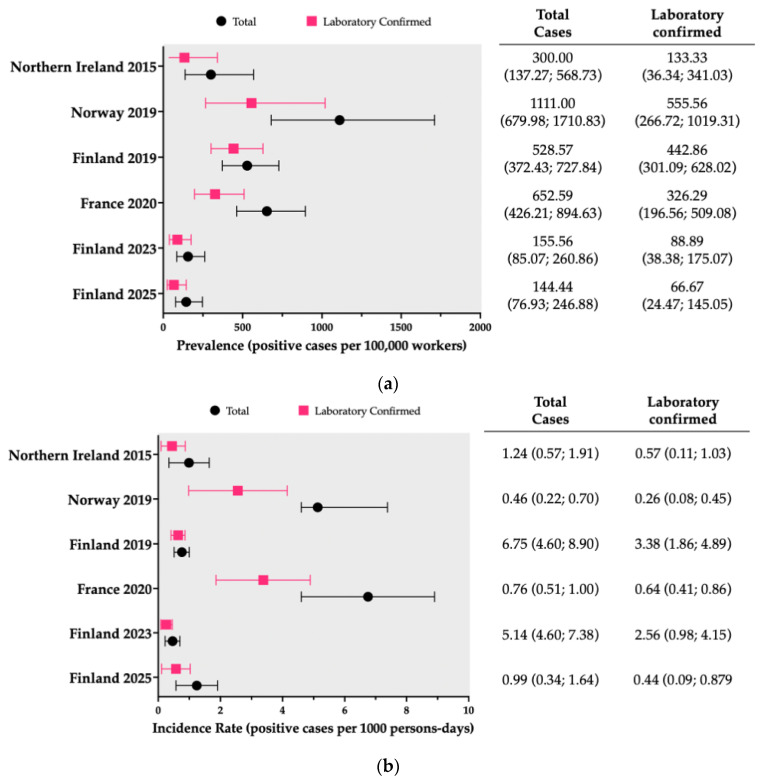
Estimates for incidence (subfigure (**a**)) and attack rates (subfigure (**b**)) in retrieved studies, calculated over the whole of reported cases and for confirmed ones [29,39,40,64,69,70,71,72,73,74].

A total of eight studies were ultimately considered consistent with the present research question [29,39,40,69,70,71,72,73] (Table 2), reporting on six outbreaks across four European countries (i.e., Northern Ireland, France, Norway, and Finland) (Figure 2), between 7 April 2015 [29,39] and 2 June 2025 (Figure 3 and Appendix Table A1) [71].

All included studies were of high quality (see Appendix Table A2 and Table A3), and their case definitions are reported in Appendix Table A4. The documented outbreaks lasted for a total of 530 days, ranging from 29 days for the 2020 outbreak in Marseille [40,69] to 209 days for the first Finnish outbreak in 2019 [70]. No clear seasonal trend was identified, as two outbreaks occurred during the winter months [40,69,73], while the remaining outbreaks were distributed throughout the remaining months of the calendar year (Appendix Table A1).

Eight reports described six distinct outbreaks; for two outbreaks (i.e., Northern Ireland 2015 and France 2020), data were consolidated from multiple sources, and each outbreak contributed a single observation to the quantitative analyses as summarized by Table 2 and Table 3 [29,39,40,69,74].

### 3.2. Clinical and Laboratory Characteristics

As summarized in Table 3, a total of 131 cases (59.5% laboratory-confirmed) were documented among 35,623 potentially exposed individuals over 108,196 person-months, corresponding to a crude incidence of 367.74 cases per 100,000 workers and 1.21 per 1000 person-months. The proportion of laboratory-confirmed cases varied across outbreaks, ranging from 44.4% in the first Belfast shipyard study (2015) [29,39] to 83.8% in the Finnish outbreak reported by Linkevicius et al. (2019) [70]. When the analysis was restricted to laboratory-confirmed cases (Figure 3), the crude incidence decreased to 218.96 cases per 100,000 workers and 0.72 per 1000 person-months. An additional case was documented from the extended report on the Northern Ireland outbreak [74], but was excluded from our estimates because the isolate was obtained from articular fluid, which was not consistent with the reporting strategies and case definitions adopted in the other studies, including earlier reports from the same site [29,39].

Overall, most cases were reported from Finland (48.9%) [70,71,72], followed by France (28.2%) [40,64,69], Norway (15.3%) [73], and Northern Ireland (6.9%) [29,39,74]. The vast majority of cases occurred in males (97.7%), with a median age ranging from 39 [40,69] to 48 years [70], and an overall range of 20 to 66 years. Regarding the clinical outcomes, 95 cases (72.5%) required hospitalization, including 17 ICU admissions (13.0%), and one death (0.8%), which was reported in the study by Linkevicius et al. 2019 [70] on the first Finnish outbreak.

Incidence rates and attack rates were calculated for the whole workforce of parent shipyards on both total and laboratory-confirmed cases and are reported in Figure 3.

Overall, the incidence of total reported cases (Figure 3a) ranged from the peak associated with outbreak from Oslo, Norway (1111/100,000 workers, 95 %CI 679.98 to 1710.83) to the lowest estimates from the more recent outbreaks in Finland (156.56/100,000 workers, 95% CI 85.07 to 260.86, and 144.44/100,000 workers, 95% CI 76.93 to 246.88 for 2023 and 2025, respectively), while the outbreak from Marseille (652.59/100,000 workers, 95% CI 426.21 to 894.63), the first outbreak from the Turku shipyard (528.57/100,000 workers, 95% CI 372.43 to 727.84), and the outbreak from Belfast (300.00/100,000 workers, 95% CI 137.27 to 568.73) scored intermediated figures. Incidence estimates of laboratory confirmed cases were similarly ranked, peaking in the Norwegian outbreak of 2019 (555.56/100,000 workers; 95% CI 266.72 to 1019.31), followed by the first Finnish outbreak of 2019 (442.86/100,000 workers; 95% CI 301.09 to 628.02), the French outbreak of 2020 (326.29/100,000 workers, 95% CI 196.56 to 509.08), the outbreak in Northern Ireland (2015) (133.33/100,000 workers, 95% CI 36.34 to 341.03), and the Finnish outbreaks of 2023 (88.89/100,000 workers, 95% CI 38.38 to 175.07) and 2025 (66.67/100,000 workers, 95% CI 24.47 to 145.05).

Attack rates (Figure 3b), for both total cases and laboratory-confirmed cases, peaked in Marseille (2020) (6.75 per 1000 person-months, 95% CI 4.60–8.90, and 3.38 per 1000 person-months, 95% CI 1.86–4.89, respectively), followed by the Norwegian outbreak (2019) (5.13 per 1000 person-months, 95% CI 4.60–7.38, and 2.56 per 1000 person-months, 95% CI 0.98–4.15). Among the Finnish outbreaks, attack rates for total cases ranged from 1.24 per 1000 person-months (95% CI 0.57–1.91) to 0.46 per 1000 person-months (95% CI 0.22–0.70), with intermediate values of 0.76 per 1000 person-months (95% CI 0.51–1.00) for the outbreaks in 2025, 2019, and 2023, respectively. Corresponding rates for laboratory-confirmed cases were 0.57 (95% CI 0.11–1.03), 0.64 (95% CI 0.41–0.86), and 0.26 (95% CI 0.08–0.45), respectively. Finally, the attack rate for the outbreak in Northern Ireland was estimated at 0.99 per 1000 person-months (95% CI 0.34–1.64) for total cases, and 0.44 per 1000 person-months (95% CI 0.09–0.87) for laboratory-confirmed cases.

**Figure 4:**
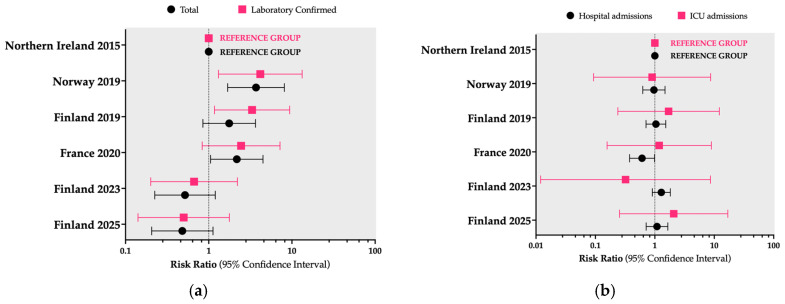
Subfigure (**a**): Estimates of the risk ratio for the occurrence of invasive pneumococcal disease, including both all cases and laboratory-confirmed cases, calculated based on the total shipyard workforce. Subfigure (**b**): Estimates of the risk ratio for hospital admissions and intensive care unit (ICU) admissions across the included studies. All calculations used the first report from Belfast, Northern Ireland (2015), as the reference group. [29,39,40,64,69,70,71,72,73,74].

As shown in Figure 4a, using the occurrence of IPD cases in Northern Ireland as the reference group and considering the entire shipyard workforce, similar proportions of documented cases were observed across all Finnish outbreaks (risk ratio [RR] 1.76, 95% CI 0.85–3.65; RR 0.52, 95% CI 0.22–1.20; and RR 0.48, 95% CI 0.21–1.13 for the 2019, 2023, and 2025 reports, respectively). In contrast, the outbreaks in Marseille (RR 2.18, 95% CI 1.05–4.49) and Oslo (RR 3.70, 95% CI 1.69–8.12) were associated with a higher occurrence of cases. When restricted to laboratory-confirmed cases, an increased occurrence was observed in the outbreaks in Oslo (RR 4.17, 95% CI 1.31–13.27) and Turku (2019) (RR 3.32, 95% CI 1.17–9.40). Nonetheless, when considering the proportions of hospital admissions and ICU admissions (Figure 4b), no substantial differences were observed across the available reports.

Main risk factors were not consistently documented across the available studies (see Appendix Table A5 for details). Nonetheless, the majority of IPD cases were reported among current or former smokers (51.5%). Alcohol consumption was recorded in only two studies, namely the reports from Kitowska et al. 2025 [72] and Manca et al. 2025 [71]. Although both reports originated from the same shipyard, alcohol consumption more frequent than once per week was reported in 9.1% of cases in the study of Kitowska et al. 2025 [72] and in 63.6% of workers from the study by Manca et al. 2025 [71].

Regarding occupational risk factors, approximately half of the cases for which data were available (44.7% of 114 workers) were exposed to welding fumes due to their daily tasks, and 36% to inorganic dusts, while smaller proportions were exposed to solvents (16.7%), and gases (2.6%). Due to inconsistent reporting of occupational titles and tasks, these data appear fragmented across studies: 43.9% of cases reportedly worked as interior outfitters, 22.8% performed daily tasks including welding and activities leading to the exposure to metal dusts, 11.4% were ship builders, 8.8% plumbers, 7.0% technicians not otherwise specified, 6.1% electricians, and 5.3% site supervisors. Notably, only the reports from the French outbreak of 2020 documented the involvement of ship crew members [40,64,69].

**Figure 5:**
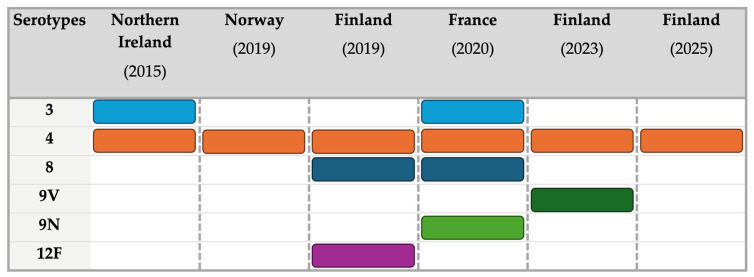
Distribution of documented serotypes across the outbreaks.

Even more limited data were available on pneumococcal serotypes, encompassing a total of 67 cases. As shown in Table 4, 67.2% of cases were associated with serotype 4, followed by serotype 12F (20.9%). Three cases (4.5%) were associated with serotype 8, two cases each with serotype 3 and 9V (3.0%), and one case with serotype 9N (1.5%) (see Appendix Table A5 for further details). As shown in Figure 5, serotype 4 was consistently reported across all the outbreaks, being the only serotype documented in Norway (2019) [73] and during the third Finnish outbreak (2025) [71].

It should be noted that the case definition implemented during the Norwegian outbreak included only serotype 4, not considering other serotypes [73]. Serotype 3 was reported in the outbreaks in Northern Ireland (2015) [29,39] and France (2020) [40,69], while serotype 8 was identified during the first Finnish outbreak (2019) [70] and in the French outbreak (2020) [40,69]. Serotypes 9V, 9N and 12F were documented in a single outbreak, namely the second Finnish outbreak (2023) [72], the French outbreak [40,69], and the first Finnish outbreak (2019) [70], respectively. Regarding coverage by available vaccines, all identified serotypes were included in PPSV23, while 98.5% were covered by PCV20, followed by PCV15 and PCV 13 (76.1%), and PCV21 (32.8%) (Appendix Table A6).

In 58 cases, sequence data on sampled pathogens were provided, and the most frequently represented genotype was Sequence Type (ST) 801 (63.8%), followed by ST 6202 (15.5%), and ST 205 (5.2%). A tentative analysis of the relationships between the documented serotypes has been provided by Gladstone et al. 2022 [66], with subsequent updates in the reports from Kitowska et al. 2025 [72] and Manca et al. 2025 [71].

### 3.3. Management of Shipyard Outbreaks

Data on the outbreak’s management were consistently available. Non-pharmaceutical interventions were deployed in all cases, including the provision of information and advice on IPD prevention (including the recommendation of pneumococcal vaccination), as well as promotion and, where possible, implementation of hygiene measures to prevent respiratory infections, such as the use of personal protective equipment (PPE) and regular hand washing. In contrast, only the management of the outbreak in Norway included specific interventions targeting hygiene measures and accommodation conditions for shipyard workers.

Regarding pharmacological interventions, i.e., the delivery of antibiotic prophylaxis and pneumococcal vaccines, a heterogeneous approach was documented. On the one hand, mass antibiotic prophylaxis for shipyard workers exposed to IPD cases was reported only in the study of Berild et al., 2020 [73]. On the other hand, vaccination catch-up campaigns were implemented in all outbreaks. In three cases, i.e., Northern Ireland 2015 [29,39], Finland 2019 [70], France 2020 [40,69], local health authorities administered pneumococcus vaccination using PPSV23, whereas conjugate vaccines were preferentially used in the remaining outbreaks [71,72,73], specifically PCV13 in Norway [73], and both PCV13 and PCV20 during the 2023 and 2025 outbreaks in Finland [71,72]. Due to the high proportion of seasonal influenza cases during the French outbreak in 2020, a seasonal influenza vaccine was also offered to shipyard workers [40,64,69].

### 3.4. Meta-Analysis of Retrieved Studies

**Figure A1:**
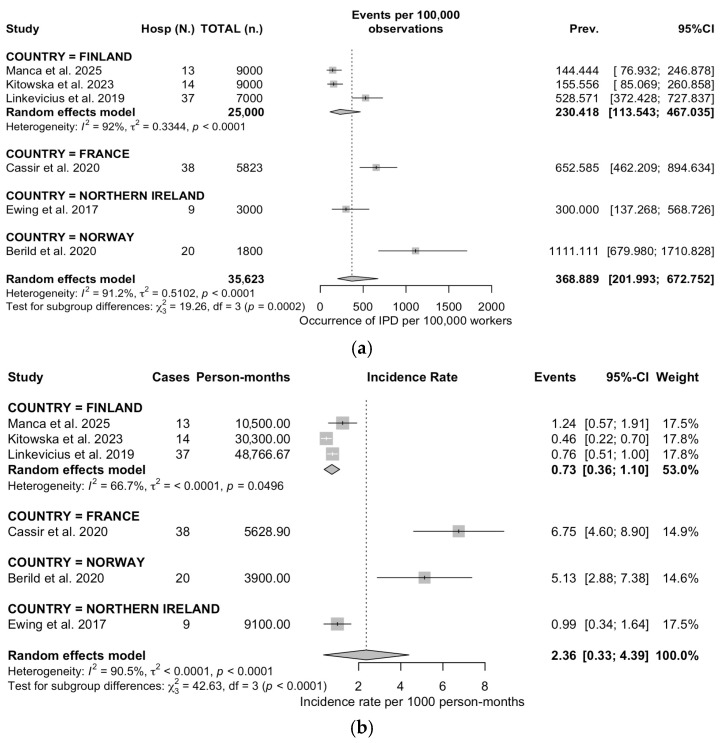
Forrest plots for the reported incidence rate of invasive pneumococcal diseases among shipyard workers as annual incidence per 100,000 workers (**a**), and incidence rate per 1000 person-months (**b**) [29,39,40,69,70,71,72].

**Figure A2:**
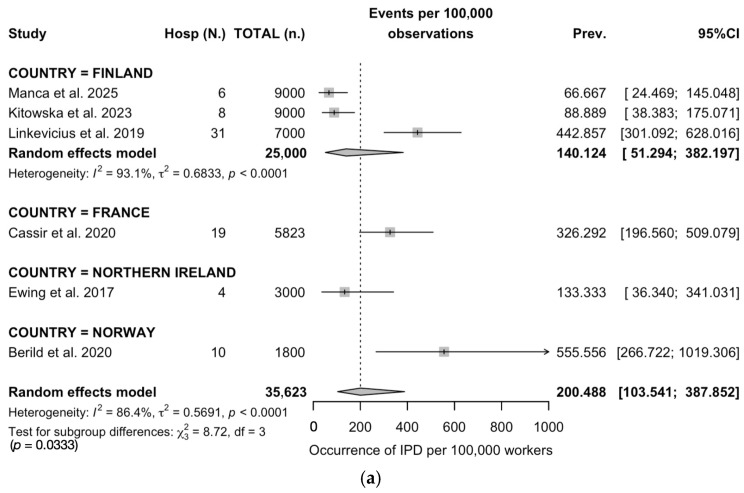

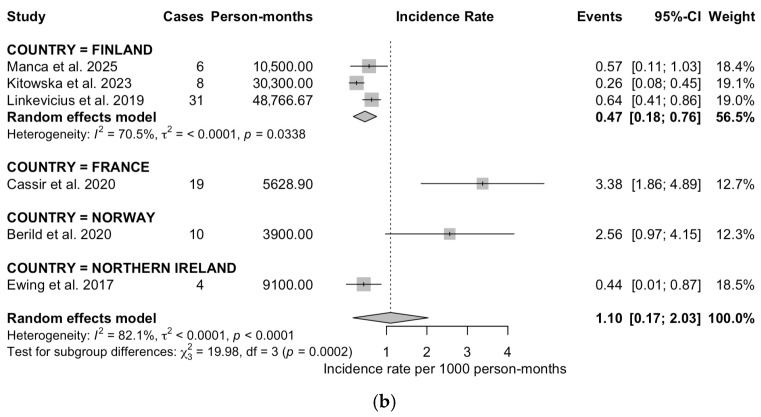
Forrest plots for the reported incidence rate of invasive pneumococcal diseases among shipyard workers as annual incidence per 100,000 workers (**a**), and incidence rate per 1000 person-months (**b**), only confirmed cases [29,39,40,69,70,71,72].

As shown in Table 4, a pooled incidence of 368.89 cases per 100,000 workers (95% CI 201.99–672.75) was estimated for total cases, compared with 200.49 cases per 100,000 workers (95% CI 103.54–387.85) for laboratory-confirmed cases. Corresponding attack rates of 2.36 cases per 1000 person-months (95% CI 0.33–4.39) and 1.10 cases per 1000 person-months (95% CI 0.17–2.03) were calculated for total and laboratory-confirmed cases, respectively. In subgroup analyses, comparisons between Finnish and non-Finnish outbreaks suggested substantial differences (see Appendix Figure A1 and Figure A2) for both incidence estimates (chi-squared test = 19.26, *p* < 0.001 and chi-squared test = 8.72, *p* = 0.033 for total and laboratory-confirmed cases, respectively) and attack rates (chi-squared test = 42.64, *p* < 0.001 and chi-squared test = 19.98, *p* < 0.001 for total and laboratory-confirmed cases, respectively).

**Figure A4:**
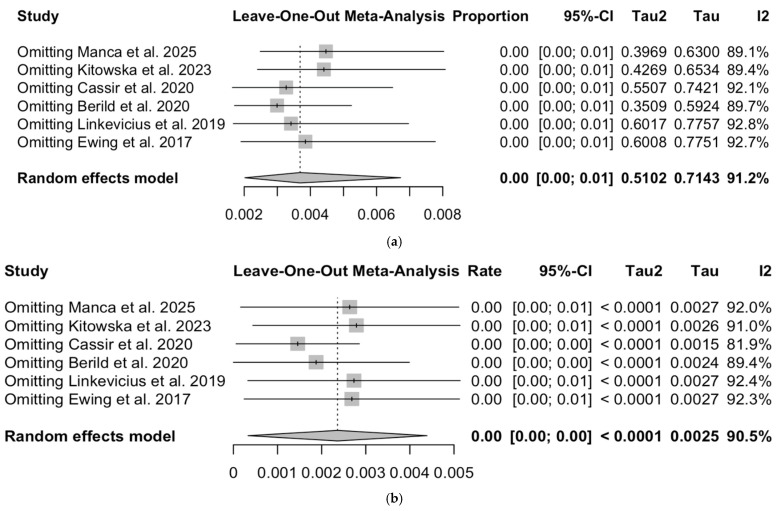
Forest plots reporting on the sensitivity analysis for: the reported incidence rate of invasive pneumococcal diseases among shipyard workers in total (**a**), and per 1000 person-months (**b**), all documented cases [29,39,40,69,70,71,72].

Focusing on hospitalization rates (see Appendix Figure A4), a pooled proportion of 79.1% (95% CI 61.9–89.8) was estimated. Again, substantial subgroup differences were identified (chi-squared test = 16.26, *p* = 0.001), with the highest estimates observed in studies from Finland (85.9%, 95% CI 75.1–92.5) and the lowest in France (47.4%, 95% CI 31.0–64.2), with intermediate estimates for Norway (75.0%, 95% CI 50.9–91.3) and Northern Ireland (77.9%, 95% CI 40.0–97.2).

Corresponding ICU admission rates and case fatality ratios were estimated at 13.7% (95% CI 8.0–20.8) and 0.8% (95% CI 0.1–5.2), respectively. National estimates for ICU admissions ranged from 10.0% (95% CI 1.2–31.7) in Norway to 15.6% (95% CI 8.6–26.7) in Finland, with intermediate values for Northern Ireland (11.1%, 95% CI 0.3–48.3) and France (13.2%, 95% CI 4.4–28.1). Reported differences were not significant in subgroup analyses (chi-squared test = 0.49, *p* = 0.992). Regarding the CFR, as the only documented death was reported in the study by Linkevicius et al. (2019) [70], a single subgroup estimate was calculated for Finland (1.6%, 95% CI 0.2 to 10.3).

All estimates on incidence and attack rates were affected by considerable heterogeneity (I^2^ > 75%). Hospitalization rates were also associated with a point estimate of I^2^ equal to 55.3% (95% CI 0.0% to 82.1%; Q = 11.19, tau^2^ = 0.625; *p* = 0.048). Although point estimates for ICU admission rates and CFR suggested an absence of substantial heterogeneity (I^2^ = 0.0% for both analyses), the corresponding 95% CI estimates of I^2^ indicated that underlying heterogeneity could not be ruled out (95% CI 0.0 to 74.6; Q = 2.08, tau^2^ < 0.001; *p* = 0.899; and 95% CI 0.0 to 74.6; Q = 0.01, tau^2^ = 0.001; *p* = 1.000, for ICU admission and case fatality ratio, respectively).

### 3.5. Publication Bias

Formal assessments of publication bias were performed but are inherently underpowered and unreliable with the small number of included studies. Visual and statistical diagnostics showed inconsistent patterns across outcomes and did not allow robust conclusions. These analyses are therefore reported as Appendix B (Appendix Figure A7, Figure A8 and Figure A9, and Appendix Table A8).

### 3.6. Sensitivity Analysis

**Figure A5:**
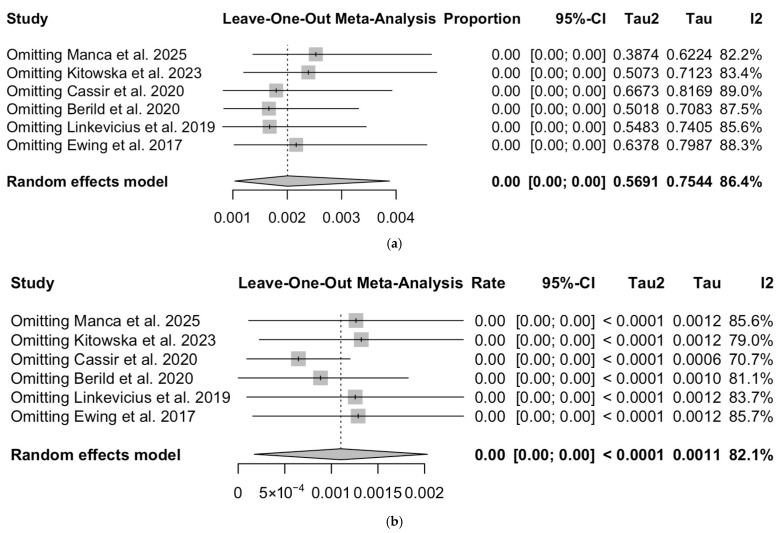
Forest plots reporting on the sensitivity analysis for: the reported incidence rate of invasive pneumococcal diseases among shipyard workers in total (**a**), and per 1000 person-months (**b**), all laboratory confirmed cases [29,39,40,69,70,71,72].

Estimates on incidence rates were noticeably affected by the removal of the studies from Cassir et al. [40,69], Berild et al. 2020 [73], and Linkevicius et al. 2019 [70]. On the contrary, the estimates on I^2^ consistently hinted towards a substantial heterogeneity of pooled incidence rates (Appendix Figure A4 and Figure A5).

**Figure A6:**
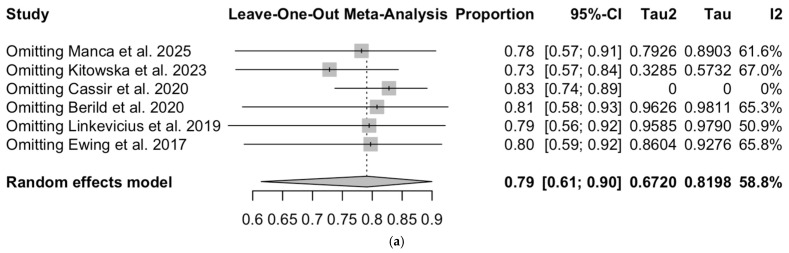

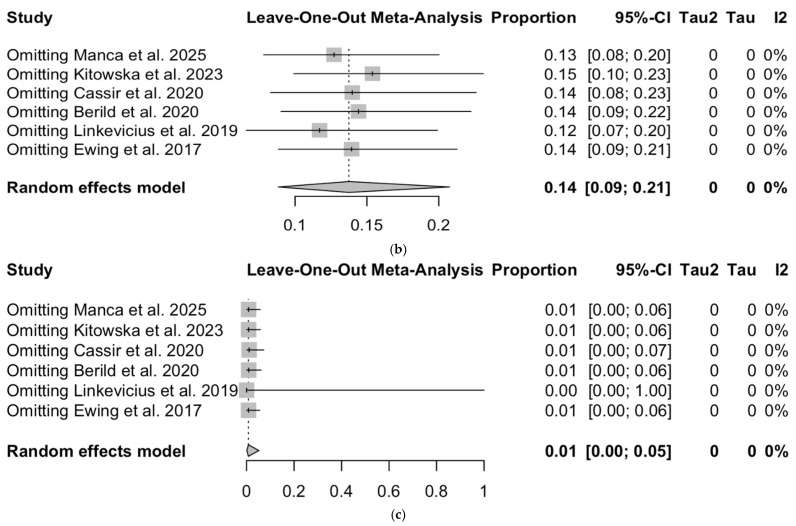
Forest plots reporting on the sensitivity analysis for: occurrence of hospitalizations among documented cases (**a**), occurrence of intensive care unit admissions (**b**), deaths (**c**) [29,39,40,69,70,71,72].

**Figure A7:**
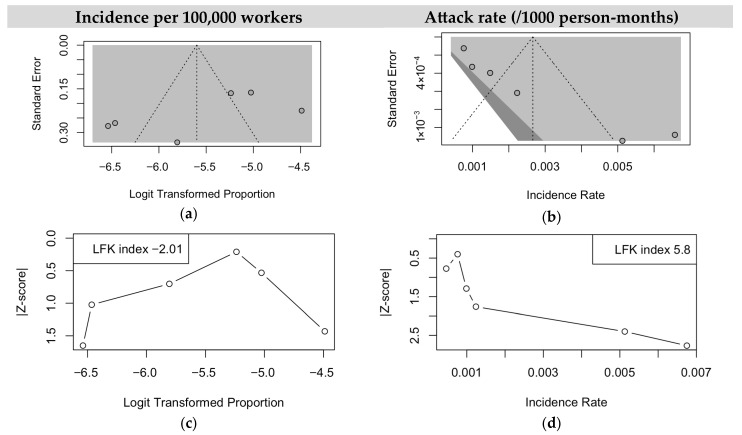

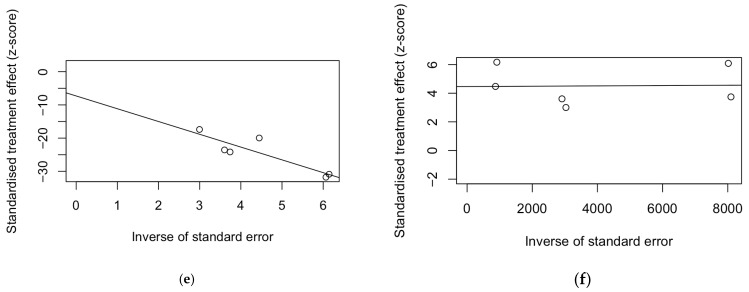
Funnel plots on the publication bias for: the reported incidence rate of invasive pneumococcal diseases among shipyard workers, reported per 100,000 workers (**a**), and as events per 1000 worker-months (**b**), and corresponding DOI plots (subfigures **c** and **d**, respectively), radial plots (subfigures **e** and **f**).

**Figure A8:**
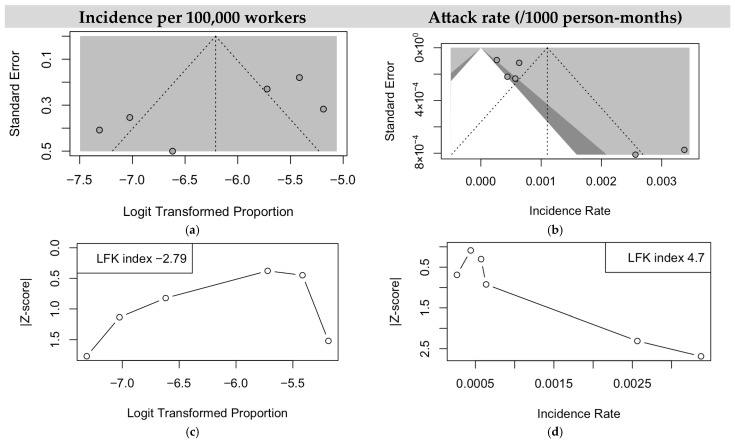

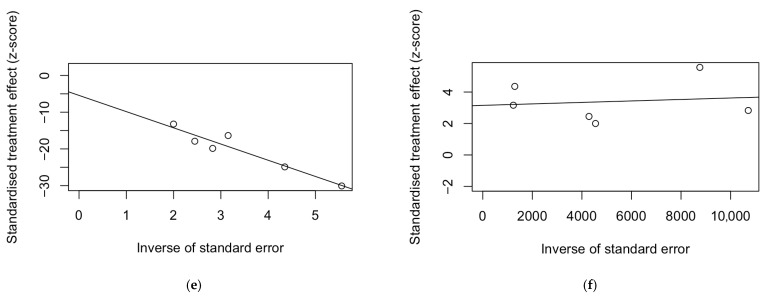
Funnel plots on the publication bias for: the reported incidence rate of invasive pneumococcal diseases among shipyard workers, reported per 100,000 workers (**a**), and as events per 1000 worker-months (**b**), only laboratory confirmed cases and corresponding DOI plots (subfigures **c** and **d**, respectively), radial plots (subfigures **e** and **f**).

**Figure A9:**
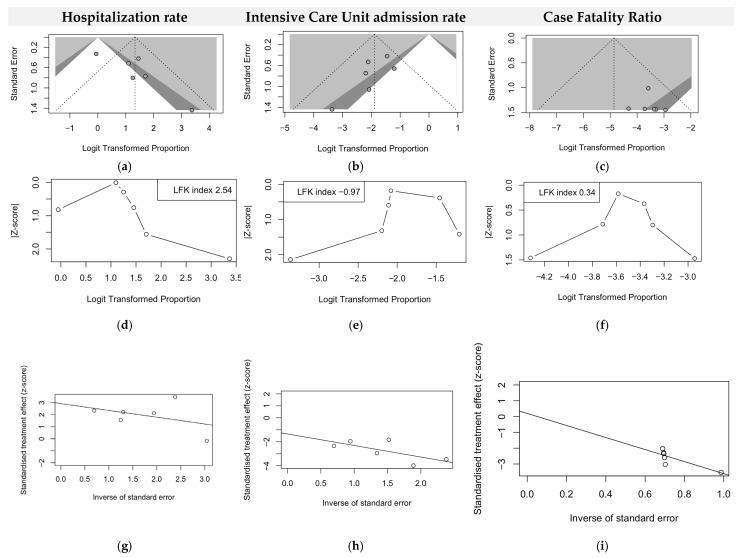
Funnel plots on the publication bias for: the reported incidence rate of invasive pneumococcal diseases among shipyard workers: occurrence of hospitalizations among documented cases (**a**), occurrence of intensive care unit admissions (**b**), deaths (**c**), corresponding DOI plots (subfigure **d**–**f**), and radial plots for exploring heterogeneity and identifying potential outliers among included studies (subfigure **g**–**i**).

When considering hospitalization rates, the exclusion of Cassir et al. [40,69] led to a substantial reduction in estimated heterogeneity, whereas the removal of Kitowska et al. (2025) [72] resulted in a substantial reduction of the pooled proportion of hospitalization (73% vs. 79.1%) (Appendix Figure A6). Finally, the removal of individual studies did not affect I^2^ estimates for ICU admissions; however, the study of Linkevicius et al. 2019 [70] influenced the pooled ICU admission rate that decreased to 12% (95% CI 7 to 20) compared to the pooled 13.1%, while the removal of Kitowska et al. [72] increased the estimate to 15% (95% CI 10 to 23).

## 4. Discussion

### 4.1. Summary of Main Findings

In the present systematic review and meta-analysis of IPD outbreaks among shipyard workers, a total of six outbreaks were documented, corresponding to nine reports [29,39,40,69,70,71,72,73,74] of very high quality. These encompassed 131 IPD cases among 35,623 workers across four sites (Belfast, Northern Ireland; Oslo, Norway; Marseille, France; and Turku, Finland) between 2015 and 2025. The corresponding incidence rates were 368.89 cases per 100,000 workers (95% CI 201.99–672.75) and 2.36 cases per 1000 person-months (95% CI 0.33–4.39) for total cases, and 200.49 cases per 100,000 workers (95% CI 103.54–387.85) and 1.10 cases per 1000 person-months (95% CI 0.17–2.03) for laboratory confirmed cases. Nearly 80% of IPD cases required hospitalization (79.1%, 95% CI 61.9–89.8), with ICU admission in 13.7% (95% CI 8.0–20.8) of cases. Only one IPD-related death was reported (CFR 0.8%, 95% CI 0.1–5.2). The most commonly identified serotype was serotype 4 (67.2% of available specimens), which was consistently reported across all included outbreaks. Approximately one quarter of cases (25.4%) reported comorbidities, particularly respiratory conditions (e.g., COPD and asthma), although individual risk factors were inconsistently reported across studies.

Regarding occupational risk factors previously associated with IPD [20,21,22,23,25,32,33,35,38,63], exposure to welding fumes and inorganic (mainly metal) dust was reported in less than half of cases (44.7% and 36.0%, respectively), while being a professional welder or performing welding-related tasks was reported in only 22.8% of cases. Notably, where data were available, findings suggested a potential role of accommodation conditions: only 20.3% of cases lived alone, whereas the remaining cases shared accommodation either with family members or with one or more roommates.

### 4.2. Generalization of Main Findings and Implications for Daily Activity

Pneumococcal infections and IPD are major causes of communicable disease morbidity [10,14,17,18], with substantial associated social and economic costs. According to the 2025 ECDC report on IPD [10], among 7000 cases with known outcome (39.5%) documented in the EU/EAA countries during 2022, 12.8% died, with higher case fatality ratios observed in older age groups (17.1% among those aged ≥ 65 years and 10.9% among those aged 45–64 years). In the present analysis, the proportion of cases requiring ICU admission was 13.0%, and the CFR was 0.8%, corresponding to a single death. While these figures appear lower than those reported in general adult populations, particularly older age groups, such comparisons should be interpreted cautiously, as they were not formally assessed and likely reflect the younger age distribution of the workforce (median age 39–48 years across studies). Moreover, the CFR estimate is based on a very small number of events and is therefore inherently unstable. Importantly, hospitalization rates remained substantial, with incidence estimates 30 to 100 times higher than those reported in the corresponding working-age groups (25–44 and 45–64 years) in the general population of the same countries during the assessed timeframes [10,17,18]. This translates into a considerable economic burden, as direct medical costs per adult IPD case have been estimated at approximately $8075 [75]. Overall, these findings suggest that, while outcomes may appear less severe than those observed in older populations, the burden of disease remains considerable, supporting the need for preventive strategies in workplace settings; in this context, the ageing European workforce could be associated with more severe outcomes in future IPD outbreaks [76]. Such strategies may include vaccination as well as non-pharmaceutical interventions (NPI).

#### 4.2.1. Vaccination Considerations and Policy Implications

The prevention of pneumococcal infection and invasive pneumococcal disease (IPD) among shipyard workers has been identified as a relevant concern for occupational health practice [20,21,22,23,25,32,33,35,38,63]. Several national bodies, including the British Department of Health, the German Ständige Impfkommission (STIKO), and the Austrian National Vaccination Plan, recommend pneumococcal vaccination for workers exposed to welding or metal fumes [77,78,79]. However, recommendations remain heterogeneous across countries [80,81], and the present study does not directly assess vaccine effectiveness, optimal strategies, or uptake in outbreak settings. Therefore, any considerations regarding vaccination should be interpreted as policy-relevant implications rather than direct evidence-based conclusions from this analysis. For example, pneumococcal vaccines are not currently included in the French official recommendations for workplace immunizations. Similarly, Italian legal framework does not formally recommend the workplace prophylaxis of IPD by means of pneumococcal vaccines, but the Italian Consolidated Act on Health and Safety at Work (Legislative Decree No. 81/2008) stresses the role of Occupational Physicians in the occupational risk assessment [82,83], as the medical professional responsible for the ultimate policy decision to recommend or not the implementation of a given vaccination in a specific occupational setting following a context-specific risk–benefit assessment.

The results from the present systematic review also share some hints on the more appropriate formulations to be recommended in daily practice. Several pneumococcal vaccines are commercially available, including PPSV and conjugate (PCV) formulations [3,4,14,84,85,86,87]. Although both PPSV and PCV vaccines have been used during outbreak management, the available data are heterogeneous and do not allow a formal comparison of their effectiveness in reducing outbreak size or severity. The distinctive properties and effectiveness profiles of PPSVs and PCVs have been discussed elsewhere [84,88,89]. Briefly, the cheaper PPSVs have an adult effectiveness estimated at around 24% (95% CI 5 to 40%) [86,90], having been considered as an effective option for healthy adults who have not completed a previous vaccination schedule with PCV [78,79,91]. However, as PPSVs are unable to elicit an effective mucosal immunity [86,90,92], their implementation does not affect the circulation of the pathogen, nor does it guarantee the actual protection of unvaccinated workers. Conjugate vaccines may therefore offer theoretical advantages, including the induction of mucosal immunity and potential reduction of transmission [93,94,95], particularly when dealing with crowded workplaces, shared households and accommodations, possibly reducing the risk of outbreaks in the specific settings of construction yards and naval shipyards [29,40,73,96]. In other words, while a vaccination strategy based on PPSVs can be considered as a rational approach for fulfilling the primary aim of the Occupational Health practice for safeguarding and promoting the health of a certain worker [97], prioritizing the use of PCV could contribute to the containment of IPD burden of disease, also benefiting individuals who are more difficult to reach through catch-up campaigns. Still, these potential features were not directly assessed in the included studies [29,39,40,69,70], and vaccination uptake was not systematically reported across studies, impairing the formal assessment of its impact on outbreak dynamics. For instance, although vaccination campaigns were rapidly and effectively organized in all Finnish outbreaks, vaccination uptake declined markedly, from over 4000 workers in 2019 [70] and over 3000 in 2023 [72] to 1500 in 2025 [71], corresponding to approximately 57.1%, 33.3%, and 16.7% of eligible workers, respectively; however, the available data did not allow the authors to assess whether these differences had any measurable impact on outbreak duration or severity.

Another issue to be considered in terms of policy is represented by vaccine formulations. PPSV23 has historically covered approximately 60–75% of adult IPD cases (see Appendix Table A6) [14,90], while earlier conjugate vaccines (e.g., PCV7) had more limited coverage [14,95], subsequently expanded in newer formulations up to PCV20 [95,98,99]. The recent introduction of PCV21 further broadens vaccine coverage by including the 21 most prevalent pneumococcal serotypes that cause IPD in adults, and therefore allows the potential replacement of PPSV23 [98], but excludes several serotypes included in PCV20.

The selection of vaccine strategies should therefore consider the serotype distribution observed in outbreaks. In the present series, the predominance of serotype 4 (67.2%) highlights the relevance of vaccines covering this serotype (PPSV23, PCV13, PCV15, PCV20), whereas PCV21 would have provided more limited coverage despite broader overall effectiveness in older populations [95,98] (see Appendix Table A6). While PPSV23 covered all detected serotypes and PCV20 omitted only one case (serotype 9N), PCV13 and PCV15 provided comparable coverage. Notably, the emergence of serotypes such as 8, 9N, and 9V in more recent outbreaks (Figure 5) further underscores the need for context-specific vaccine selection. In the end, it should be stressed that these considerations are based on descriptive serotype distributions and should not be interpreted as evidence of differential vaccine effectiveness in outbreak prevention.

Overall, vaccination represents a plausible and policy-relevant preventive strategy in high-risk occupational settings; however, the present findings do not allow direct assessment of the comparative effectiveness of specific vaccine formulations or strategies. Further studies are needed to evaluate optimal vaccination approaches in these contexts.

#### 4.2.2. Non-Pharmaceutical Interventions

Non-pharmaceutical interventions are recognized as public health measures, apart from vaccination and pharmacological treatment, that individuals and communities can adopt to help slow the spread of infectious diseases by preventing and/or controlling pathogen transmission within the community [100,101]. These interventions have become more widely recognized, even in non-healthcare settings, following the SARS-CoV-2 pandemic. In this context, it is noteworthy that during the pandemic—particularly in its early and more severe phases, when NPIs were more widely and strictly implemented as the primary available preventive option—no further IPD outbreaks were documented, even though shipyard and naval construction activities were reduced but not completely halted [102]. The significance of NPI in the management of IPD outbreaks also stresses the role of environmental factors in the spread of the pathogen across workplaces, particularly across a workforce characterized by an unresolved continuum between occupational settings and non-occupational human interactions. Even though the chronic damage of upper and lower airways associated with the exposure to welding fumes represents a likely explanation for the increased occurrence of IPD in certain occupational settings [20,24,31,32], outbreaks of IPD have been previously described in settings such as mines and extractive industry where the exposures to welding fumes are limitedly documented, but the workforce shares very similar specificities in terms of health status and housing issues [103,104]. In this regard, it is important to emphasize that while NPIs were consistently implemented across all documented outbreaks, only the Norwegian outbreak included a specific intervention aimed at improving hygiene and living conditions of shipyard workers as a measure to interrupt transmission chains of the involved pneumococcal strains [73]. The promotion of healthier and safer workplaces in shipyard settings would therefore benefit not only from direct interventions, including the implementation of NPIs even in non-pandemic contexts, but also from ensuring improved accommodation conditions for the workforce, as previously documented in the management of SARS-CoV-2 outbreaks in the meat processing industry in 2020 and 2021 [105,106,107].

#### 4.2.3. Health Promotion Across the Workplaces

Considerable evidence indicates that the health status of workers employed in shipyard and naval construction settings is affected by a high prevalence of behavioral risk factors, including smoking and excessive alcohol consumption. In our sample, the pooled prevalence of smoking history (current and former) involved approximately half of cases, and about one third reported regular alcohol consumption (see also Appendix Table A5). Although inconsistent reporting across the included studies limits a more detailed analysis of individual risk factors, the available evidence suggests a high prevalence of established risk factors for IPD, including obesity, diabetes mellitus, and chronic respiratory conditions [8,9,63,108,109]. Therefore, the promotion of healthy lifestyles—including balanced nutrition, regular physical activity, weight control, and avoidance of tobacco and alcohol—not only contributes to the prevention of major non-communicable diseases (such as cardiovascular disease, diabetes, and cancer) and their risk factors (e.g., hypertension, hyperglycemia, and overweight), but may also reduce the risk of invasive infections, including IPD, and their more severe complications [110]. In other words, comprehensive efforts to prevent IPD in high-risk shipyard settings may represent a case study of how workplace safety and health strategies should address not only the prevention of occupational hazards, but also the promotion of physical health and overall well-being through a holistic “Total Worker Health^®^ (TWH)” approach, as first proposed by the US National Institute for Occupational Safety and Health in 2011 [111].

### 4.3. Limits of the Present Study

Despite the potential interest for Public Health and Occupational Health professionals, our study is affected by some limitations.

First and foremost, the quality of the original studies severely affects that of secondary studies, including meta-analyses [57,112]. Even though the gathered studies appear to be of relatively high or even very high quality, the estimates were based on a relatively reduced number of populations, highly selected: as a consequence, the potential generalizability of data drawn from retrieved observational studies could be questioned, as otherwise stressed by hints for publication and small study effect, particularly when dealing with the outcomes of IPD.

A second limitation is the small number of included outbreaks and events. Accordingly, the meta-analysis was undertaken as an exploratory synthesis of patterns across outbreaks. The substantial heterogeneity observed limits the interpretability of pooled estimates, which should be viewed as descriptive summaries rather than precise effect measures, reflecting greater between-outbreak variability than within-study precision. Subgroup analyses were similarly underpowered. Therefore, the findings should be regarded as hypothesis-generating.

Third, although the characteristics of invasive pneumococcal disease (IPD) are well established by public health authorities [10,17,18], the analysis and comparison of case definitions across the included studies indicate that reporting heterogeneity may have affected our pooled estimates. Case definitions differed in several key aspects. First, the Norwegian outbreak definition focused exclusively on serotype 4 cases [73], and therefore non–serotype 4 infections may have been missed, potentially leading to under-ascertainment. Second, while most studies restricted case identification to shipyard workers, the report by Cassir et al. [40,69] also included crew members. Although this broader inclusion supports a role for environmental factors in outbreak propagation, it may have diluted the contribution of occupational exposures, such as welding and metal fumes. Third, even within the same setting (i.e., the Turku shipyard), consistent case definitions were not applied across investigations [70,71,72]. More recent reports, such as Manca et al. 2025 [71], incorporated nucleic acid amplification tests and radiological criteria for lobar pneumonia, which were not used in earlier investigations. This evolution in diagnostic criteria may have resulted in differential ascertainment, with potential over-ascertainment in more recent studies or, conversely, under-ascertainment in earlier ones. Overall, these discrepancies likely resulted in both under- and over-ascertainment of cases, thereby limiting the comparability of incidence and attack-rate estimates across outbreaks. To partially address this issue, we conducted a sensitivity analysis restricted to laboratory-confirmed IPD cases, which yielded lower incidence estimates. However, this approach increases specificity at the expense of sensitivity and does not fully account for differences in diagnostic intensity across studies. Therefore, heterogeneity in case definitions represents a major source of bias, and our findings should be interpreted with caution.

Fourth, as stressed by Gladstone et al. [66], and subsequently reinstated by more recent reports from Finnish outbreaks [71,72], the documented outbreaks were extensively associated with the clonal expansion of a certain strain of serotype 4. It follows that the analysis presented here may have limited generalizability beyond documented substrains of serotype 4. On the other hand, due to the unpredictability of the emergence of new clones with outbreak potential, the lessons learned by the retrospective analysis of available data may be of critical importance for the implementation of appropriate preventive measures and avoiding future occupational outbreaks.

A further limitation relates to the use of the total shipyard workforce as the denominator for incidence calculations, which implicitly assumes homogeneous exposure across workers. On the contrary, shipyard workforces are highly heterogeneous and dynamic, with substantial short-term variability in workforce size and composition [37,39,41,66,69,70,72,73,74]. Moreover, different professional groups do not share the same exposure profiles [37,41,69,113]: for example, workers directly involved in welding or operating within shipyard facilities or inside vessels may have substantially different exposure compared with administrative staff or support personnel, who may share only limited common environments (e.g., canteens) or none. Therefore, the use of aggregate workforce figures likely introduces exposure misclassification and may dilute incidence estimates, leading to underestimation of risk among truly exposed workers. Notably, several included studies reported only approximate or coarse estimates of workforce size, further limiting the precision of the denominators. Therefore, the reported incidence figures should be interpreted as crude and indicative measures rather than accurate estimates of risk in specific occupational subgroups.

## 5. Conclusions

In conclusion, this systematic review identified a series of IPD outbreaks occurring in shipyard settings, predominantly in Europe. Although most cases were linked to the clonal expansion of a single serotype (notably serotype 4), workplace conditions and workforce characteristics likely contributed to the observed high incidence rates. Despite this, clinical outcomes were associated with low rates of intensive care unit admission and a case fatality ratio below 1%, findings that could be tentatively explained by the relatively young age of the affected workforce rather than by specific pathogen-related factors. Nevertheless, the availability of effective preventive strategies—ranging from improvements in accommodation and lifestyle conditions to vaccination—highlights the need for a proactive and prevention-oriented role of occupational physicians in the management of shipyard workers.

## Figures and Tables

**Table 1:** Detailed search strategy by database source and number of retrieved entries.

Source	Search Strategy	No. of Entries
Pubmed	(“*Streptococcus pneumoniae*” OR “pneumococcus” OR “pneumococcal infection”) AND (“meningitis” OR “pneumonia” OR “bacteremia” OR “invasive pneumococcal disease” OR “IPD”) AND (“occupation*” OR “work-related” OR “worker*” OR “job” OR “shipyard” OR “dock” OR “dockyard”)	129
medRxiv		41
Scopus		524
EMBASE	(‘streptococcus pneumoniae’/exp OR ‘streptococcus pneumoniae’ OR ‘pneumococcal infection’) AND (‘invasive pneumococcal infection’ OR ‘meningitis’ OR (OR ‘pneumonia’ OR ‘IPD’ OR ‘’bacteremia’) AND (‘occupation’ OR ‘work’ OR ‘workforce’) AND (‘shipyard’ OR ‘dock’ OR ‘dockyard’)	976

**Table 2:** Characteristics of outbreak studies included in the systematic review (Note: ICU = intensive care; 95% CI = 95% confidence interval).

Study	Country	Outbreak Period, Days (n/530; %)	Population at Risk (N/35,623; %)	No. Cases		Age (Years; Median, Range)	Males (n/TOT; %)	Hospital Admissions (n/TOT, %)		Deaths (n/TOT.; %)	Serotypes (n.; %)
				TOT. (n/N, %)	Laboratory Confirmed (n/TOT; %)			Total	ICU		
Manca et al. 2025 [71]	Finland	28/04/2025 02/06/2025 (35, 6.6%)	9000 (25.3%)	13, 0.3%	6, 46.2%	43 (40, 58)	13, 100%	11, 84.6%	3, 23.1%	0, -	4 (4, 100%)
Kitowska et al. 2025 [72]	Finland	19/08/2023 28/11/2023 (101, 19.1%)	9000 (25.3%)	14, 0.5%	8, 57.1%	42 (39, 50)	13, 92.9%	14, 100%	0, -	0, -	4 (5, 71.4%) 9V (2, 28.6%)
Cassir et al. 2020 [40] Cassir et al. 2021 [69]	France	09/01/2020 07/02/2020 (29, 5.5%)	5823 (16.4%)	38, 0.6%	19, 51.4%	39 (22; 66)	37, 97.4%	18, 47.4%	5, 13.2%	0, -	3 (1, 11.1%) 4 (5, 55.6%) 8 (2, 22.2%) 9N (1, 11.1%)
Linkevicius et al. 2019 [70]	Finland	03/05/2019 28/11/2019 (209, 39.4%)	7000 (19.6%)	37, 0.5%	31, 83.8%	48 (37; 55)	36, 97.3%	30, 81.1%	7, 18.9%	1, 2.7%	12F (14, 53.8%) 4 (11, 42.4%) 8 (1, 3.8%)
Berild et al. 2020 [73]	Norway	28/01/2019 03/04/2019 (65, 12.3%)	1800 (8.0%)	20, 1.1%	10, 50%	47 (20; 60)	20, 100%	15, 75.0%	2, 10.0%	0, -	4 (17, 100%)
Ewing et al. 2017 [29] Patterson et al. 2015 [39]	Northern Ireland	07/04/2015 07/07/2015 (91, 19.1%)	3000 (8.4%)	9, 0.3%	4, 44.4%	43 (20; 60)	9, 100%	7, 77.8%	1, 11.1%	0, -	3 (1, 25.0%) 4 (3, 75.0%)

**Table 3:** Main characteristics of the pooled sample.

Variable	N	n/N (%)
Total cases	131	131 (100%)
Confirmed cases	131	78 (59.5%)
Countries		
Finland	131	64 (48.9%)
France	131	37 (28.2%)
Norway	131	20 (15.3%)
Northern Ireland	131	9 (6.9%)
Age (years)		
Range of medians	131	39 to 48
Range (min.–max.)	131	20 to 66
Male gender	131	128 (97.7%)
Current/Former smoker	131	67 (51.1%)
Alcohol consumption more than once in a week	22	8 (36.4%)
Reporting any comorbidity	114	29 (25.4%)
Accommodation		
Living alone	64	13 (20.3%)
With other individuals (no family)	64	28 (43.8%)
With other individuals (family)	64	23 (35.9%)
Shared accommodation		
1 roommate	51	14 (27.5%)
2 roommates or more	51	25 (49.0%)
Occupational tasks		
Welder/exposed to welding fumes	114	26 (22.8%)
Interior outfitters/installers	114	50 (43.9%)
Ship Builders	114	13 (11.4%)
Plumbers	114	10 (8.8%)
Technicians	114	8 (7.0%)
Electricians	114	7 (6.1%)
Site supervisors	114	6 (5.3%)
Exposure to		
Welding fumes	114	51 (44.7%)
Inorganic dusts	114	41 (36.0%)
Gasses	114	3 (2.6%)
Solvents	114	19 (16.7%)
Serotype		
3	67	2 (3.0%)
4	67	45 (67.2%)
8	67	3 (4.5%)
9V	67	2 (3.0%)
9N	67	1 (1.5%)
12F	67	14 (20.9%)
Included in		
PPSV23	67	67 (100%)
PCV13	67	51 (76.1%)
PCV15	67	51 (76.1%)
PCV20	67	66 (98.5%)
PCV21	67	22 (32.8%)
Genotypes		
ST 66	58	1 (1.7%)
ST 205	58	3 (5.2%)
ST 239	58	1 (1.7%)
ST 801	58	37 (63.8%)
ST 1220	58	1 (1.7%)
ST 1280	58	1 (1.7%)
ST 1460	58	1 (1.7%)
ST 1480	58	1 (1.7%)
ST 2025	58	1 (1.7%)
ST 6202	58	9 (15.5%)
ST 15063	58	2 (3.4%)
Vaccination status available	131	6 (4.6%)
Management of the outbreak		
Antibiotic therapy	6	1 (16.7%)
Vaccination	6	6 (100%)
Conjugated vaccines	6	3 (50.0%)
Polysaccharide vaccine	6	3 (50.0%)
Hospital admissions	131	95 (72.5%)
ICU admissions	131	17 (13.0%)
Deaths	131	1 (0.8%)

**Table 4:** Summary of pooled estimates for incidence rate (per 100,000 workers and per 1000 person-months), hospitalization rate, intensive care unit (ICU) admission rate, and case fatality ratio (CFR) calculated by means of random-effects meta-analysis with their corresponding 95% confidence intervals.

	Estimate (95% CI)	I^2^ (95% CI)	Q	tau^2^	*p* Value
Incidence per 100,000 workers					
Total cases	368.89 (201.99; 672.75)	91.2% (83.6; 95.3)	56.66	0.510	<0.001
Laboratory confirmed cases	200.49 (103.54; 387.85)	86.4% (72.5; 93.2)	36.71	0.754	<0.001
Attack rate (per 1000 person-months)					
Total cases	2.36 (0.33; 4.39)	90.5% (82.0; 95.0)	52.50	<0.001	<0.001
Laboratory confirmed cases	1.10 (0.17; 2.03)	82.1% (61.9; 91.6)	27.92	<0.001	<0.001
Hospitalizations (cases per 100 IPD)	79.1% (61.9; 89.8)	55.3% (0.0; 82.1)	11.19	0.625	0.048
ICU admissions (cases per 100 IPD)	13.7% (8.0; 20.8)	0.0% (0.0 to 74.6)	2.08	<0.001	0.899
CFR (cases per 100 IPD)	0.8% (0.1; 5.2)	0.0% (0.0 to 74.6)	0.01	0.001	1.000

**Figure A3:**
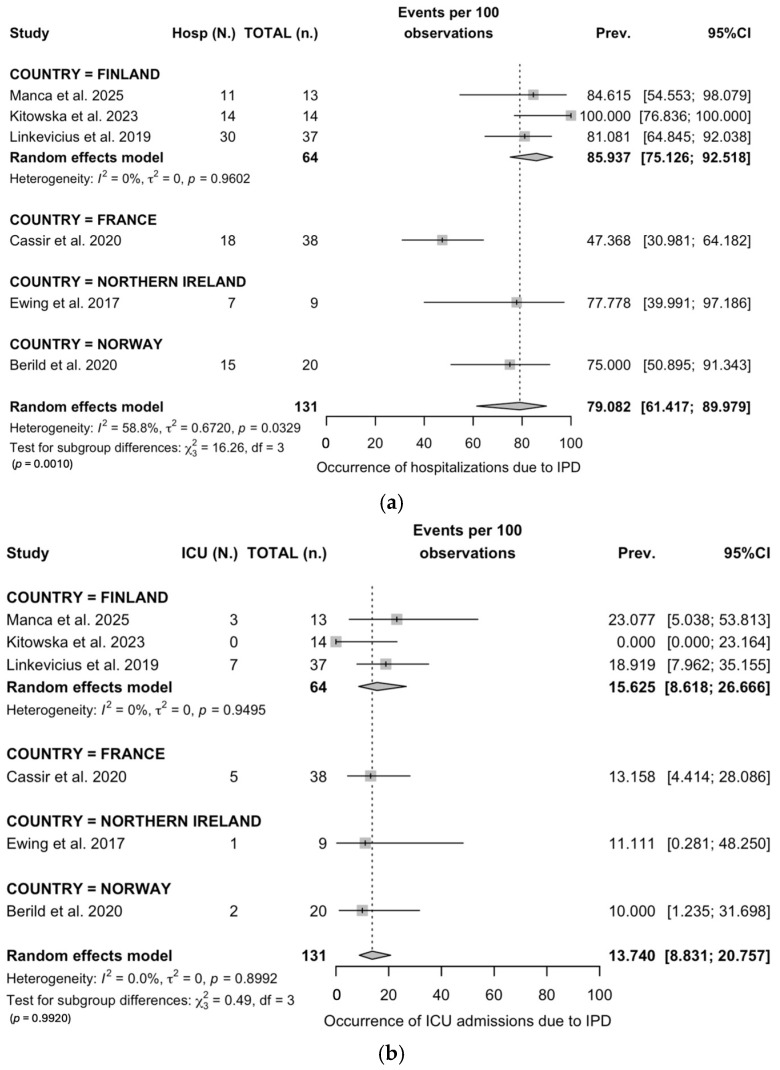

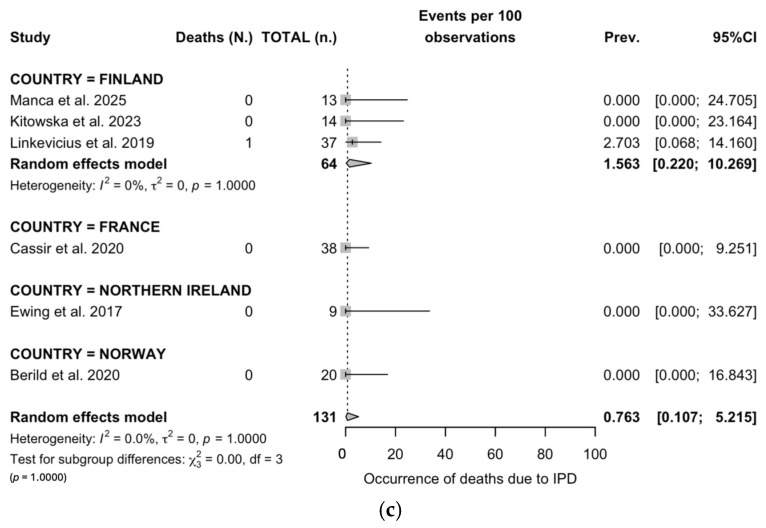
Forrest plots for hospitalization rates (**a**), intensive care unit (ICU) admission rates (**b**), and case fatality ratio (CFR) (**c**) for studies on invasive pneumococcal diseases outbreaks among shipyard workers [29,39,40,69,70,71,72].

## Data Availability

Raw data are available in the Supplementary Material.

## Supplementary Materials

The following supporting information can be downloaded at: https://www.mdpi.com/article/10.3390/vaccines14050437/s1, PRISMA checklist of the present study; raw data included in the analyses.

## Abbreviations

The following abbreviations are used in this manuscript:

CFR	Case Fatality Ratio
COPD	Chronic Obstructive Pulmonary Disease
df	Degrees of Freedom
ECDC	European Center for Disease Prevention and Control
ICU	Intensive Care Unit
IPD	Invasive Pneumococcal Disease
LFK	Luis Furuya-Kanamori
NOS	Newcastle–Ottawa Scale
OR	Odds Ratio
PCV	Pneumococcal Conjugate Vaccine
PECO	Patient/Population/Problem; Exposure; Control/Comparator; Outcome
PPE	Personal Protective Equipment
PPSV	Pneumococcal Polysaccharide Vaccine
REM	random effect model
REML	restricted maximum likelihood
RR	Risk Ratio
95%CI	95% Confidence Interval

## Appendix A

**Table A1:** Occurrence of documented outbreaks included in the present study by calendar months; grey cells = month characterized by documented cases.

Outbreak	Jan.	Feb.	Mar.	Apr.	May.	Jun.	Jul.	Aug.	Sep.	Oct.	Nov.	Dec.
N. Ireland 2015												
Norway 2019												
Finland 2019												
France 2020												
Finland 2023												
Finland 2025												

**Table A2:** A summary of the risk of bias assessment according to the Newcastle–Ottawa quality assessment scale for included studies (* = item positively checked; - = item negatively checked).

Study	Selection			Comparability		Outcome			Total
	Representativeness	Exposed Cohort	Ascertainment	Result Not Present at the Start of the Study	Comparability for Confounders	Assessment of the Outcome	Follow-Up Duration	Adequacy of Follow-Up	N/9
Manca et al. 2025 [71]	*	*	*	*	**	*	*	*	9/9
Kitowska et al. 2025 [72]	*	*	*	*	**	*	*	*	9/9
Cassir et al. 2021 [69]	*	*	*	*	**	*	*	-	8/9
Cassir et al. 2020 [40]	*	*	*	*	*	*	*	-	7/9
Linkevicius et al. 2019 [70]	*	*	*	*	**	*	*	*	9/9
Berild et al. 2020 [73]	*	*	*	*	*	-	*	*	7/9
Ewing et al. 2017 [29]	*	*	*	*	*	*	*	*	9/9
Patterson et al. 2015 [39]	*	*	*	*	*	*	*	-	7/9

**Table A3:** Summary of the quality appraisal of included studies by means of an adapted version of the “Outbreak Reports and Intervention studies Of Nosocomial infection (ORION)” statement [48,49].

Item	Study							
Citation	Patterson et al. 2015 [39]	Ewing et al. 2017 [29]	Berild et al. 2020 [73]	Linkevicius et al. 2019 [70]	Cassir et al. 2020 [40]	Cassir et al. 2021 [69]	Kitowska et al. 2025 [72]	Manca et al. 2025 [71]
Setting (country, site)	Northern Ireland (Belfast)	Northern Ireland (Belfast)	Norway (Oslo)	Finland (Turku)	France (Marseille)	France (Marseille)	Finland (Turku)	Finald (Turku)
Outbreak period	07/04/2015 07/07/2015	07/04/2015 07/07/2015	28/01/2019 03/04/2019	03/05/2019 28/11/2019	09/01/2020 07/02/2020	09/01/2020 07/02/2020	19/08/2023 28/11/2023	28/04/2025 02/06/2025
Population (denominator)	3000	3000	1800	7000	5823	5823	9000	9000
1. Background and objectives								
Clear rationale for the outbreak report	2	2	2	2	2	2	2	2
Explicit objectives/research questions	2	2	2	2	2	2	2	2
Description of setting and context	2	2	1	2	2	2	2	2
Outbreak Description & Case Definition								
Clear case definition (clinical/lab/epi)	1	1	2	2	2	2	2	2
Consistency of case definition over time	2	2	2	2	2	2	2	2
Description of case finding (active/passive)	1	1	2	2	2	2	2	2
Completeness of case ascertainment discussed	1	1	1	2	2	2	2	2
Epidemiological Methods								
Study design clearly stated (descriptive/analytic)	2	2	2	2	2	2	2	2
Population at risk clearly defined	2	2	2	2	2	2	2	2
Timeframe adequately specified	2	2	2	2	2	2	2	2
Use of appropriate epidemiological measures	2	2	1	2	2	2	2	2
Handling of confounding/bias (if analytic)	2	2	2	2	2	2	2	2
Microbiological & Laboratory Data								
Laboratory confirmation methods described	1	1	2	2	2	2	2	2
Typing/genotyping reported (if relevant)	2	2	2	2	2	2	2	2
Consistency of diagnostic methods	2	2	2	2	2	2	2	2
Environmental & Occupational Assessment								
Description of environmental conditions	0	0	0	2	0	2	2	2
Description of occupational exposures	2	2	1	2	2	2	2	2
Assessment of transmission pathways	1	1	2	2	2	2	2	2
Control Measures & Interventions								
Control measures clearly described	2	2	2	2	1	2	2	2
Timing of interventions reported	2	2	2	2	2	2	2	2
Vaccination strategies described	2	2	2	2	2	2	2	2
Evaluation of intervention effectiveness	2	2	2	2	2	2	2	2
Outcomes								
Clinical outcomes reported	2	2	1	2	2	2	2	2
Completeness of outcome data	0	1	1	2	1	2	2	2
Follow-up duration adequate	0	1	2	2	0	1	2	2
Data Analysis & Reporting								
Statistical methods appropriate	2	2	2	2	1	1	2	2
Uncertainty reported (CI, variability)	1	1	2	2	1	1	2	2
Transparency of data sources	2	2	2	2	2	2	2	2
Bias & Limitations								
Discussion of selection bias	2	2	2	2	1	2	2	2
Discussion of information bias	2	2	2	2	2	2	2	2
Limitations clearly acknowledged	1	1	2	2	2	2	2	2
Ethical & Reporting Considerations								
Ethical considerations reported	2	2	2	2	2	2	2	2
Transparency and reproducibility	2	2	2	2	2	2	2	2
Total (n/66, %)	53, 80%	55, 83%	58, 88%	66, 100%	57, 86%	63, 95%	66, 100%	66, 100%
Quality (High ≥ 75%; Moderate: 50–74%; Low: < 50%)	High	High	High	High	High	High	High	High

Note: each item was scored as follows: 0 = No/not reported; 1 = Partially reported; 2 = Adequately reported; NA = Not applicable.

**Table A4:** Summary of case definition.

Condition	Northern Ireland 2015 [29,39,74]	Norway 2017 [73]	Finland 2019 [70]	France 2020 [40,69]	Finland 2023 [72]	Finland 2025 [71]
**Confirmed case**	An individual who has worked at the Belfast shipyard since 19 January 2015 and presents with a clinical diagnosis of IPD or pneumococcal pneumonia AND at least one of the following: (1) pneumococcus isolated from normally sterile site (blood, cerebrospinal fluid (CSF), joint, peritoneum, pleural fluid or other, but not sites such as eye); (2) pneumococcal DNA or antigen detected in fluid from a normally sterile site: (3) pneumococcal antigen detected in urine.	Invasive pneumococcal disease with serotype 4 isolated from a normally sterile site.	An individual who had worked at the shipyard after 1 February 2019 and presented with a clinical diagnosis consistent with IPD OR pneumococcal pneumonia AND had *Streptococcus pneumoniae* isolated from blood or cerebrospinal fluid or pneumococcal antigen detected in urine	A worker or a crew member of the cruise liner in the shipyard of Marseille with a clinical and radiological diagnosis of pneumonia from 3 January 2020 AND *Streptococcus pneumoniae* isolated from blood or endobronchial samples OR pneumococcal antigen detected in the urine	An individual with a clinical presentation consistent with pneumococcal pneumonia or IPD who was working in Turku Shipyard and was diagnosed after 1 August 2023 AND *Streptococcus pneumoniae* was isolated from blood or cerebrospinal fluid OR *Streptococcus pneumoniae* antigen was detected in urine.	An individual with a radiologically confirmed lower tract infection compatible with signs of pneumococcal pneumonia, such as lobar pneumonia, diagnosed since 15 April 2025 and up to 2 months after the last case was diagnosed, and who had worked in the Turku shipyard since 1 April. AND microbiological confirmation through culture OR nucleic acid amplification detection of *Streptococcus pneumoniae* from blood AND/OR a positive pneumococcal urinary antigen test.
**Probable case**	An individual who has worked at the Belfast shipyard since 19 January 2015 and presents with a clinical diagnosis of IPD or pneumonia (supported by radiographic imaging) where serious pneumococcal disease based on available clinical, microbiological and epidemiological evidence is the most likely diagnosis, in the absence of laboratory confirmation.	worked at the specific shipyard AND had a clinical presentation compatible with lower respiratory tract infection or IPD, but without microbiological confirmation OR serotype 4 isolated from a non-sterile medium (e.g., nasopharyngeal swab or sputum culture).	An individual who had worked at the shipyard after 1 February 2019 and presented with a clinical diagnosis consistent with IPD without laboratory confirmation.	A worker or a crew member of the cruise liner in the shipyard of Marseille with a clinical and radiological diagnosis of pneumonia from 3 January 2020 without laboratory confirmation	An individual with a clinical presentation consistent with pneumococcal pneumonia or IPD who was working in Turku Shipyard and was diagnosed after 1 August 2023 without laboratory confirmation	An individual with a radiologically confirmed lower tract infection compatible with signs of pneumococcal pneumonia, such as lobar pneumonia, diagnosed since 15 April 2025 and up to 2 months after the last case was diagnosed, and who had worked in the Turku shipyard since 1 April without laboratory confirmation
**Possible case**	An individual who has worked at the Belfast shipyard since 19 January 2015 and presents with a clinical diagnosis of IPD or pneumococcal pneumonia (supported by radiographic imaging) where diagnoses other than serious pneumococcal disease are at least as likely.					

**Table A5:** Characteristics of individual risk factors from retrieved outbreaks.

Condition	Northern Ireland 2015 [29,39]	Norway 2017 [73]	Finland 2019 [70]	France 2020 [40,69]	Finland 2023 [72]	Finland 2025 [71]
Total cases (TOT)	9	20	37	38	14	13
Documented cases (N/TOT, % on total)	9 (100%)	20 (100%)	25 (67.6%)	38 (100%)	11 (78.6%)	11 (84.6%)
Occupational status (n/N, %)						
Welders	3 (33.3%)	10 (50.0%)	4 (16.0%)	1 (2.6%)	4 (36.4%)	4 (36.4%)
Technicians	1 (11.1%)	-	-	7 (18.4%)	-	-
Metal workers	1 (11.1%)	-	-	-	-	-
Interior outfitters/installers	-	10 (50.0%)	16 (64.0%)	6 (15.8%)	9 (81.8%)	9 (81.8%)
Plumbers	-	-	3 (12.0%)	-	-	7 (63.6%)
Electricians	-	-	7 (28.0%)	-	-	-
Painters	-	-	-	1 (2.6%)	-	-
Carpenter	-	-	-	1 (2.6%)	-	1 (9.1%)
Crew member	-	-	-	4 (10.5%)	-	-
Site supervisors/managers	1 (11.1%)	-	3 (12.0%)	2 (5.3%)	-	-
Fire guards	-	-	-	2 (5.3%)	-	-
Ship builders	-	-	2 (8.0%)	-	-	11 (100%)
Documented exposures to (n/N, %):						
Metal fumes	6 (66.6%)	10 (50.0%)	15 (60.0%)	13 (34.2%)	10 (90.9%)	7 (63.6%)
Inorganic dusts	-	-	22 (88.0%)	8 (21.1%)	11 (100%)	-
Solvents	-	-	14 (56.0%)	5 (13.2%)	-	-
Gases	-	-	3 (12.0%)	-	-	-
Borrowing PPE	-	-	-	-	2 (18.2%)	1 (9.1%)
Coinfections (in general)	-	-	-	19 (50.0%)	-	-
Coinfections (seasonal influenza)	-	-	-	6 (15.8%)	-	-
Comorbidities (n/N, %)						
Any	1 (11.1%)	1 (5.0%)	3 (12.0%)	17 (44.7%)	3 (27.3%)	4 (36.4%)
COPD	-	-	1 (4.0%)	4 (10.5%)	-	-
Asthma	-	-	1 (4.0%)	-	-	-
Diabetes	-	-	-	1 (2.6%)	-	-
Cardiovascular diseases	-	1 (5.0%)	-	2 (5.3%)	-	-
Lung cancer	-	-	1 (4.0%)	-	-	-
Alcohol (>1 once a week)	-	-	-	-	1 (9.1%)	7 (63.6%)
Delivered vaccines						
PPSV23	X		X	X		
PCV13		X			X	X
PCV20					X	X
Seasonal flu			X			
Non Pharmacological Interventions						
Information and advice on IPD	X	X	X	X	X	X
Hygiene measures for prevention of respiratory diseases (PPE, hand washing)	X	X	X	X	X	X
Hygiene measures on accommodations	-	X	-	-	-	-

**Table A6:** Serotypes of retrieved cases compared to the coverage of main available vaccines (i.e., PPSV23 = Pneumococcal polysaccharide vaccine; PCV Pneumococcal conjugate vaccine).  = included;  = not included.

Serogroup	Available Vaccines							Isolates from Shipyard Outbreaks (N/67, %)
	PPSV23	PCV7	PCV10	PCV13	PCV15	PCV20	PCV21	
1								
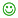
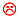
2								
3								2, 3.0%
4								45, 67.2%
5								
6A								
6B								
7F								
8								3, 4.5%
9N								1, 1.5%
9V								2, 3.0%
10A								
11A								
12F								14, 20.9%
14								
15A								
15B								
15C								
16F								
17F								
18C								
19A								
19F								
20A								
22F								
23A								
23B								
23F								
24F								
31								
33F								
35B								

**Table A7:** Comparison of incidence rates per 100,000 people from the outbreaks included in the analyses, and by age groups 25–44 and 45–64 years in the parent country during the corresponding timeframe. Data were obtained from available reports of European Centers for Disease Prevention and Control [10,17,18].

Outbreak	Country	Reported Incidence	Incidence in the Parent Country	
			Age Group 25–44 Years	Age Group 45–64 Years
		n./100,000 People	n./100,000 People	n./100,000 People
Belfast, 2015 [29,39,74]	Northern Ireland (UK)	300.00	3.31	8.15
Oslo, 2017 [73]	Norway	1111.11	3.21	11.44
Turku, 2019 [70]	Finland	528.57	5.84	16.02
Marseille, 2020 [40,64,69]	France	652.58	2.00	4.23
Turku, 2023 [71,72]	Finland	155.56	2.78	6.70
Turku, 2025 [71]	Finland	144.44	2.78	6.70

## Appendix B. Analysis of Publication Bias

Publication bias was initially assessed by calculating funnel plots for the assessed outcomes. Briefly (Appendix Figure A7 and Figure A8), all funnel plots were asymmetrical, suggesting the likelihood that pooled data were affected by a substantial publication bias.

However, calculation of Doi plots and corresponding LFK indexes hinted at the presence of some degree of publication bias only for incidence rates when calculated in terms of events per 1000 person-months (LFK index = 5.08), but also when calculated for 100,000 persons (LFK index = −2.01), and hospitalization rate (LFK index = 2.54), while it was substantially ruled out for ICU admission and case fatality ratio (LFK index = −0.97, and LFK index = 0.34, respectively).

Consistent with calculation of LFK index, estimates of Egger’s test on incidence rates per 1000 person-months (t = 4.43, Bias 4.465, SE = 1.008, *p* = 0.011) and hospitalization rates (t = 2.38, Bias 3.010, SE 1.265, *p* = 0.076), suggested an underlying publication bias (Appendix Table A8) that was otherwise ruled out for incidence per 100,000 workers (t = −1.57, Bias −7.300, SE 4.647, *p* = 0.191), as well as for ICU admission and case fatality ratio estimates (*p* = 0.139 and *p* = 0.866, respectively), although the results should be interpreted with caution given the limited number of included studies.

Eventually, radial plots (scatter plots of standardized estimates) were calculated for studying the corresponding small-study bias. Plots calculated for incidence estimates and attack rates (Appendix Figure A7), hospitalization rate, and ICU admission (Appendix Figure A8) were characterized by a scattered distribution of the point estimates across the regression lines, ruling out that the estimates may have been somehow affected by smaller samples. On the contrary, estimates on CFR were unevenly distributed across the regression line, but the resulting estimates were possibly affected by the low number of episodes (Appendix Figure A9).

**Table A8:** Summary of Egger’s test results on the main findings reported in this meta-analysis on invasive pneumococcal disease among shipyard workers.

Finding	t	df	Bias (SE)	tau^2^	*p*-Value
Incidence (per 100,000 per year)					
Laboratory confirmed cases	−2.19	4	−5.429 (2.475)	4.167	0.093
Total cases	−1.57	4	−7.300 (4.647)	8.760	0.191
Incidence (per 1000 person-months)					
Laboratory confirmed cases	−2.97	4	3.153 (1.061)	2.175	0.041
Total cases	4.43	4	4.465 (1.008)	2.223	0.011
Hospitalization (per 100 workers)	2.38	4	3.010 (1.265)	1.596	0.076
ICU admission (per 100 workers)	−1.84	4	−1.295 (0.702)	0.401	0.139
Case fatality ratio (per 100 workers)	0.18	4	0.188 (1.044)	0.136	0.866
