# Supplementary material for: A Possible Unmet Need: Pneumococcal Vaccination in the Workplaces—A Systematic Review of Invasive Pneumococcal Disease Among Shipyard Workers
Source: Vaccines (Basel). 2026 May 13;14(5):437. doi: 10.3390/vaccines14050437 (PMC13211396; doi:10.3390/vaccines14050437)
Supplement: Supplementary file 1 [file vaccines-14-00437-s001.zip › vaccines-4285179-supplementary/pneumo2026.pdf]

Variable			Study					
			**Ewing et al. 2015**	**Berild et al. 2019**	**Linkevicius et al. 2020**	**Cassir et al. 2020**	**Kitowska et al. 2023**	**Manca et al. 2025**
			Settings					
**Country**	No. of countries: 4 (Finland, France, Northern Ireland, Norway)		Northern Ireland (UK)	Oslo, Norway	Turku, Finland	Marseille, France	Turku, Finland	Turku, Finland
**Timespan**	First episode Last episode	07/04/2015 02/06/2025	07/04/2015 07/07/2019	28/01/2019 03/04/2019	03/05/2019 28/11/2019	09/01/2020 07/02/2020	19/08/2023 28/11/2023	28/04/2025 02/06/2025
**Total length of the outbreak**	days	No./530 (%)	91 (19.1%)	65 (12.3%)	209 (39.4%)	29 (5.5%)	101 (19.1%)	35 (6.6%)
**Exposed workers**	Total shipyard workforce	No./22,423 (%)	3,000 (13.4%)	1,800 (8.0%)	5,000 (22.3%)	5,823 (26.0%)	2,800 (12.5%)	4,000 (17.8%)
			**Characteristics of cases**					
**Total cases**	No./130 (%)		9 (6.9%)	20 (15.4%)	37 (28.5%)	37 (28.5%)	14 (10.8%)	13 (10.0%)
**Confirmed cases**	78/130 (60.0%)	No./78 (%)	4 (5.1%)	10 (12.8%)	31 (39.7%)	19 (24.4%)	8 (10.3%)	6 (7.7%)
**Hospitalized**	98/130 (75.4%)	No./97 (%)	7 (7.2%)	15 (15.5%)	30 (30.9%)	18 (18.6%)	14 (14.4%)	13 (13.4%)
**Pneumonia**	46/46 (100%)	No./46 (%)	9 (19.6%)	n.a.	n.a.	37 (80.4%)	n.a.	n.a.
**ICU**	17/116 (14.7%)	No./17 (%)	1 (5.9%)	2 (11.8%)	7 (41.2%)	4 (23.5%)	n.a.	3 (17.6%)
**Deaths**	1/130 (0.8%)	No./1 (%)	0 (-)	0 (-)	1 (100%)	0 (-)	0 (-)	0 (-)
			**Demographics**					
**Age (years)**	Median (range)		43 (20, 60)	47 (20, 60)	48 (37, 55)	39 (22, 66)	42 (39, 50)	43 (40, 58)
**Males**	127/130 (97.7%)	No./127 (%)	9 (7.1%)	20 (15.7%)	36 (28.3%)	36 (28.3%)	13 (10.2%)	13 (10.2%)
**Females**	3/130 (2.3%)	No./3 (%)	0 (-)	0 (-)	1 (33.3%)	1 (33.3%)	1 (33.3%)	0 (-)
			**MIcrobiological features**					
**Serotype**								
**12F**	14/67 (20.9%)	No./14 (%)	0 (-)	0 (-)	14 (100%)	0 (-)	0 (-)	0 (-)
3	2/67 (3.0%)	No./2 (%)	1 (50.0%)	0 (-)	0 (-)	1 (50.0%)	0 (-)	0 (-)
4	45/67 (67.2%)	No./45 (%)	3 (6.7%)	17 (37.8%)	11 (24.4%)	5 (11.1%)	5 (11.1%)	4 (8.9%)
8	3/67 (4.5%)	No./3 (%)	0 (-)	0 (-)	1 (33.3%)	2 (66.7%)	0 (-)	0 (-)
9V	2/67 (3.0%)	No./2 (%)	0 (-)	0 (-)	0 (-)	0 (-)	2 (100%)	0 (-)
9N	1/67 (1.5%)	No./1 (%)	0 (-)	0 (-)	0 (-)	1 (100%)	0 (-)	0 (-)
**Genotype**								
**66**	1/58 (0.8%)	No./1 (%)	0 (-)	0 (-)	0 (-)	1 (100%)	0 (-)	0 (-)
**205**	3/58 (2.3%)	No./3 (%)	1 (33.3%)	0 (-)	0 (-)	2 (66.7%)	0 (-)	0 (-)

Variable			Study					
			Ewing et al. 2015	Berild et al. 2019	Linkevicius et al. 2020	Cassir et al. 2020	Kitowska et al. 2023	Manca et al. 2025
**239**	1/58 (0.8%)	No./1 (%)	0 (-)	0 (-)	0 (-)	0 (-)	1 (100%)	0 (-)
**801**	37/58 (28.5%)	No./37 (%)	2 (5.4%)	15 (40.5%)	7 (18.9%)	4 (10.8%)	5 (13.5%)	4 (10.8%)
**1220**	1/58 (0.8%)	No./1 (%)	0 (-)	0 (-)	0 (-)	1 (100%)	0 (-)	0 (-)
**1280**	1/58 (0.8%)	No./1 (%)	0 (-)	0 (-)	0 (-)	1 (100%)	0 (-)	0 (-)
**1460**	1/58 (0.8%)	No./1 (%)	0 (-)	0 (-)	0 (-)	1 (100%)	0 (-)	0 (-)
**1480**	1/58 (0.8%)	No./1 (%)	0 (-)	0 (-)	1 (100%)	0 (-)	0 (-)	0 (-)
**2025**	1/58 (0.8%)	No./1 (%)	0 (-)	0 (-)	0 (-)	0 (-)	1 (100%)	0 (-)
**6202**	9/58 (6.9%)	No./9 (%)	0 (-)	0 (-)	9 (100%)	0 (-)	0 (-)	0 (-)
**15063**	2/58 (1.5%)	No./2 (%)	0 (-)	2 (100%)	0 (-)	0 (-)	0 (-)	0 (-)
**Risk factors**								
**Current / former smoker**	67/130 (51.5%)	No./67 (%)	6 (9.7%)	7 (11.3%)	29 (46.8%)	14 (14.5%)	7 (11.3%)	4 (6.5%)
**Living conditions**								
**Alone**	13/64 (20.3%)	No./13 (%)	n.a.	n.a.	7 (53.8%)	n.a.	3 (23.1%)	3 (23.1%)
**With roommates**	28/64 (43.8%)	No./27 (%)	n.a.	n.a.	14 (50.0%)	n.a.	8 (28.6%)	6 (21.4%)
**1 roommate**	14/51 (27.5%)	No./14 (%)	n.a.	n.a.	7 (50.0%)	n.a.	7 (50.0%)	n.a.
**2 roomates or more**	25/51 (49.0%)	No./25 (%)	n.a.	n.a.	21 (84.0%)	n.a.	4 (16.0%)	n.a.
**Occupational tasks**								
**Welder / exposed to welding fumes**	36/110 (32.7%)	No./36 (%)	9 (25.0%)	"most"	15 (41.7%)	2 (5.6%)	4 (11.1%)	4 (11.1%)
**Vaccination status available**	6/130 (4.6%)	No./6 (%)	0 (-)	0 (-)	0 (-)	0 (-)	5 (83.3%)	1 (16.7%)
**Intervention**								
**Antibiotherapy**	1 out of 6 outbreaks (16.7%)		-	Azythromicin	-	-	-	-
**Vaccine**	PCV13: 3 out of 6 outbreaks (50.0%)<br>PPV23: 3 out of 6 outbreaks (50.0%)		PPV23	PCV13	PPV23	PPV23	PCV13	PCV13
